# Evaluating High-Throughput *Ab Initio* Gene Finders to Discover Proteins Encoded in Eukaryotic Pathogen Genomes Missed by Laboratory Techniques

**DOI:** 10.1371/journal.pone.0050609

**Published:** 2012-11-30

**Authors:** Stephen J. Goodswen, Paul J. Kennedy, John T. Ellis

**Affiliations:** 1 School of Medical and Molecular Sciences, and the Ithree Institute at the University of Technology Sydney (UTS), New South Wales, Australia; 2 School of Software, Faculty of Engineering and Information Technology and the Centre for Quantum Computation and Intelligent Systems at the University of Technology Sydney (UTS), New South Wales, Australia; University of Rome, Italy

## Abstract

Next generation sequencing technology is advancing genome sequencing at an unprecedented level. By unravelling the code within a pathogen’s genome, every possible protein (prior to post-translational modifications) can theoretically be discovered, irrespective of life cycle stages and environmental stimuli. Now more than ever there is a great need for high-throughput *ab initio* gene finding. *Ab initio* gene finders use statistical models to *predict* genes and their exon-intron structures from the genome sequence alone. This paper evaluates whether existing *ab initio* gene finders can effectively predict genes to deduce proteins that have presently missed capture by laboratory techniques. An aim here is to identify possible patterns of prediction inaccuracies for gene finders as a whole irrespective of the target pathogen. All currently available *ab initio* gene finders are considered in the evaluation but only four fulfil high-throughput capability: AUGUSTUS, GeneMark_hmm, GlimmerHMM, and SNAP. These gene finders require training data specific to a target pathogen and consequently the evaluation results are inextricably linked to the availability and quality of the data. The pathogen, *Toxoplasma gondii*, is used to illustrate the evaluation methods. The results support current opinion that predicted exons by *ab initio* gene finders are inaccurate in the absence of experimental evidence. However, the results reveal some patterns of inaccuracy that are common to all gene finders and these inaccuracies may provide a focus area for future gene finder developers.

## Introduction

Discovering novel proteins that are potentially expressed by a pathogen in a host is still a major challenge facing the scientific community. The laboratory approach for discovering proteins is hindered by restrictions and limitations, such as: some pathogens are difficult and/or dangerous to cultivate in the laboratory; the expression of proteins may be different *in vitro* than those proteins expressed during infection *in vivo*; the expression of proteins may be different at various stages of the life cycle of the pathogen or under altered environmental conditions; and there is a bias towards abundant proteins that are more easily identified in the laboratory [1].

Discovering proteins is further challenged by the fact the literature now conclusively shows that a single gene produces multiple protein isoforms under varying conditions, which drastically increases the scale and scope of the proteins to discover [2]. Experimentally creating all the relevant conditions to capture the entire complement of proteins is beyond current technology. Currently, the laboratory approach can only capture a subset of an unknown sized proteome. It is expected that many important proteins are eluding the capture and consequently are absent from further downstream analysis. Even RNA-seq – a revolutionary tool for transcriptome analysis [3], which undoubtedly will discover novel proteins and protein isoforms – is still faced with the same laboratory restrictions and limitations in capturing mRNA.

An *in silico* approach has the potential to capture the proteins missed during the laboratory discovery process. It is well-known that the nuclear genome of a pathogen encodes the entire repertoire of genes, which potentially can express as proteins. Theoretically, therefore, every possible protein (prior to post-translational modifications) can potentially be discovered, irrespective of life cycle stages and environmental stimuli, by unravelling the code within the genome. The falling cost and on-going improvements in next generation sequencing technology is advancing sequencing of genomes at an unprecedented level. It is not inconceivable to expect that in the very near future we will readily have available a genome sequence for any strain that has been isolated *and* have multiple genome sequences of the same strain. Now more than ever there is a great need for high-throughput *ab initio* gene finding in order to mine this data for genes.

Research into identifying genes in anonymous genomic sequences has been going on for more than 20 years and there is an abundance of literature [4], [5], [6], [7], [8], [9], [10]. Gene finding has proved to be an immense challenge [11]. On one hand there is practically a universal pattern to the exon-intron structure of eukaryotic genes but on the other hand the pattern creates much ambiguity. Most eukaryotic genes begin with a start codon of ATG and end with one of three possible stop codons – TAG, TAA, or TGA. Non-ATG start codons do exist and are discussed in the literature [12], [13], [14], but they are comparatively rare in eukaryotes. There are no known non-standard stop codons in eukaryotic genomes although, as a rare exception, the relatively uncommon amino acid selenocysteine is encoded by the stop codon TGA [15]. Embedded between the start and stop codons are zero or more introns (non-coding regions), which are delimited by donor sites (typically GT) and acceptor sites (typically AG) [16]. There are a few cases of splice sites in the literature [17], [18] that deviate from the typical GT-AG. The ambiguity occurs because not all ATGs in a DNA sequence code for a start codon, and not all GTs and AGs are splice sites. Thus, unlike the actual spliceosome of a eukaryotic cell, no algorithm as yet can precisely recognise exon-intron structures from DNA sequence alone. The best achievable approach from an *ab initio* perspective is to use statistical models to *predict* genes and their exon-intron structures. There are numerous gene prediction programs freely available which attempt to meet the challenge. At the time of writing, more than 60 could be found in the literature though many of the published URLs were no longer valid. Table 1 shows a small sample of the most common gene finders. The disparity in their gene predictions for the same DNA sequence shows that the gene finding challenge is still to be overcome [10].

**Table 1:** Gene finders in chronological order based on release year.

Year	Gene Finder Name	Type++	Comments
1991	GRAIL [19]	*Ab initio*	No longer supported
1992	GeneID [20]	*Ab initio*	
1993	GeneParser [21]	*Ab initio*	
1994	Fgeneh [22]	*Ab initio*	Finds single exon only
1996	Genie [23]	Hybrid	
1996	PROCRUSTES [24]	Evidence based	
1997	Fgenes [25]	Hybrid	No download version
1997	GeneFinder	*Ab initio*	Unpublished work
1997	GenScan [26]	*Ab initio*	
1997	HMMGene [27]	*Ab initio*	No download version
1997	GeneWise [28]	Evidence based	
1998	GeneMark.hmm [29]	*Ab initio*	
2000	GenomeScan [30]	Comparative	
2001	Twinscan [31]	Comparative	
2002	GAZE [32]	Comparative	
2004	Ensembl [33]	Evidence based	
2004	GeneZilla/TIGRSCAN [34]	*Ab initio*	No longer supported
2004	GlmmerHMM [34]	*Ab initio*	
2004	SNAP [9]	*Ab initio*	
2006	AUGUSTUS+ [35]	Hybrid	
2006	N-SCAN [36]	Comparative	
2006	Twinscan_EST [37]	Comparative+Evidence	
2006	N_Scan_EST [37]	Comparative+Evidence	
2007	Conrad [38]	*Ab initio*	
2007	Contrast [39]	*Ab initio*	
2009	mGene [40]	*Ab initio*	No longer supported

++ Hybrid = *ab inito* and evidence based; Comparative = genome sequence comparison.

The representation of all eukaryotic pathogens at the genetic level is structured the same i.e. a series of four nucleotides with motif signals (start and stop codon, and donor and acceptor sites) defining the coding exons. Since most of the popular *ab initio* gene finders work within this universal gene structure and take into account the pathogen’s compositional (codon) biases, it is not the type of pathogen that mostly affects the accuracy of the predictions. Irrespective of the pathogen, gene finders can have a better or worse accuracy depending on the quality of input and training data [41]. For example, a gene finder always performs better when using a model trained on a target rather than a foreign pathogen [9].

In addition to *ab initio* (or intrinsic) [6], there are two other methods to computational gene finding – evidence based (or extrinsic) [42], and genome sequence comparison [31]. Strictly evidence based gene finders use evidence such as DNA copies of mRNA (cDNA) and/or proteins and/or expressed sequence tags (ESTs) [16]. They work by aligning evidence sequences, ideally from different types, to the pathogen genome based on sequence similarity. In effect the evidence constitutes combined exons and the alignment to genome attempts to reintroduce introns into the evidence to determine the exon-intron structure of the gene. Evidence based gene finders are ideal for genomic annotation but have limited value for finding novel genes. Aligning an mRNA or a protein sequence (which has been translated back to nucleotides) to a genome provides no additional evidence that it is a novel protein (this is not surprising since they *are* in themselves the evidence for a potential novel protein irrespective of any alignment). Programs such as BLASTP (if given protein evidence) and BLASTX (if given mRNA evidence) to find homologs would be more appropriate than a gene finder to determine if an mRNA or protein is novel. Genome sequence comparison exploits the use of sequence conservation to help in identify coding exons. The underlying principle of the method is to compare anonymous genomic sequences from the same or different organisms, under the assumption that regions conserved in high complexity sequences will tend to correspond to coding exons from homologous genes [43]. In other words, the conserved regions between related organisms are more likely to be coding, and conversely the divergent regions more likely to be non-coding. Genome sequence comparison has the potential to discover novel genes and is becoming a more feasible method owing to the increasing availability of genome sequences [44].

This paper explores whether existing bioinformatics tools can efficiently discover pathogen proteins missed by laboratory techniques and in effect describes an evaluation of publicly available gene finders when used in the absence of experimental evidence. *Toxoplasma gondii*, which is an apicomplexan pathogen responsible for birth defects in humans [45], was the chosen species to illustrate the evaluation methods and to compare the performance of the gene finders. This pathogen was particularly chosen because it is an important model system for the phylum Apicomplexa [46], [47], [48] and has experimentally validated data that can be used for training and testing. All apicomplexans differentiate to forms that invade single or multiple hosts to complete extremely complex life cycles. For example, *Toxoplasma gondii* can infect almost any tissue of warm blooded animals [49] and possesses several life cycle stages yet to be completely characterised at the proteome and transcriptome level. Experimentally creating all the relevant conditions, from which to capture the entire complement of expressed proteins, is beyond current technology. Consequently, there are novel proteins encoded in apicomplexan genomes that have presently missed capture by laboratory techniques, which theoretically could be captured by *ab initio* gene finders. An important aim here is to identify possible patterns of inaccuracies that are common to all evaluated gene finders irrespective of the pathogen, such as: are the start and/or stop exons predicted less accurately than internal exons? Does the distance between exons affect accuracy? Is there any difference in accuracy between forward and reverse strand predictions? Knowing common inaccuracies may provide a focus area for future gene finder developers.

## Methods

There were seven specific tasks undertaken to complete the overall evaluation of the gene finders: 1) selecting the appropriate gene finders for evaluation, 2) collating a validated dataset and creating training models specific to each gene finder, 3) evaluating the prediction accuracies using sensitivity and specificity measures, 4) determining how well the gene finders perform in locating genes by aligning predicted and test sequences, 5) evaluating at the protein level, 6) classifying the predicted gene locations relative to test genes, and 7) finding potential novel genes.

### Selecting the Gene Finders for Evaluation

The initial challenge was determining which gene finder programs, from so many, to include in the evaluation. To narrow down the number of candidates, there were six important criteria used to assess the inclusion or exclusion of a program – public availability, operating platform, high-throughput functionality, cell type, training data, and software support. Each criterion is now described in more detail: 1) Public availability – the program had to be freely downloadable and have standalone capability; 2) Type of operating platform – the numerous programs potentially available can be classified into three platform categories: web interface, Microsoft Windows, and Linux. The web interface programs are by far the most prevalent due to their immediate accessibility (i.e. no installation) and ease of use. However, processing enormous amounts of input to find genes on a genome wide scale is currently unproductive through web interfaces. Only Linux supported programs were chosen because Linux is becoming an international standard for academia and research; 3) High- throughput functionality – the programs needed to process large numbers of input in a timely manner. What constitutes processing completion in a ‘timely manner’ is debatable. Most of the standalone programs trialled gave no indication of progress when executed. Here, if the command prompt was not returned within 48 hours it was assumed the program was in a loop (hanging) and subsequently excluded from the selection process; 4) Cell type – only programs specific to eukaryotic organisms were used; 5) Training data – the program had to either provide a readymade trained model or functionality to create one for the target pathogen; and 6) Maintained and supported – ideally the program should have documentation, contact support for bug fixes and enhancements, and most importantly work consistently without errors. This criterion was only partly fulfilled for most programs. So in summary the programs chosen for evaluation were standalone programs for eukaryotes that could be freely downloaded, executed in a Linux environment, enable high-throughput processing, and have either a readymade trained model or functionality to create one for the target pathogen.

Four *ab initio* gene finder programs fulfilled the selection criteria–GeneMark.hmm [29], AUGUSTUS [35], [50], SNAP [9], and GlimmerHMM [34], [51]. The programs GeneZilla [34], mGene [40] (uses machine learning techniques), and Conrad [38] (based on conditional random fields) were tested but not included for evaluation due to consistent undocumented program crashes. All the evaluated gene finders use a variation of hidden Markov models (HMMs) [52]. A HMM is used to statistically model structure of DNA sequences and each gene finder has its own complex internal algorithm to decode the HMM into gene predictions [16]. The Supporting Information S1 provides detailed information about these gene finders including download URLs, and basic background on gene prediction and HMMs. For specific details on how these programs work refer to the following references [9], [29], [34], [35]. All evaluated gene finders run in a command-line mode and to effectively run them it was required to know for each program the format of the input files, the command-line parameters (as they vastly impact the output), and the type of output to expect. For brevity, the commands and parameters used for setting up and running the programs are also in Supporting Information S1.

### Creating Training and Validated Datasets

The fundamental method for evaluating gene finders is comparing predicted genes with validated genes at the nucleotide, exon, and gene level [8]. Finding experimentally validated genes and extracting the exons is not a straightforward task. The method used to obtain gene sequences and their exon locations for the evaluation is described in Supporting Information S1. A validated gene set was created which comprised all genes from the *T. gondii* genome that have evidence for protein expression based on mass spectrometry analyses. These genes (3,432 in total) were downloaded from ToxoDB database [53]. The ToxoDB database is a central depository specific to various types of T. *gondii* biological data and can be found at http://toxodb.org/toxo/. All downloaded genes were checked to confirm expected start codon (ATG), stop codon (TAG, TGA, or TAA), donor (GT) and acceptor (AG) consensus sequences. Any genes not meeting this expectation were removed from the validation set. There were five exceptions for the start and stop codons, no exceptions for acceptor sequences, and 129 for donor sequences. However, these 129 genes uniformly had GC as the donor sequence and were not removed on the assumption that this alternative donor consensus (as in non-canonical splice site) was not a manifestation due to sequencing errors. A check to see if there were any redundant genes was also conducted. If two genes from the same chromosome had a 95% or greater similar coverage the gene with the smallest query length was removed from the validated set. No redundant genes were found. Several genes had 100% coverage to genes from a different chromosome. These genes were not removed.

**Figure 1:**
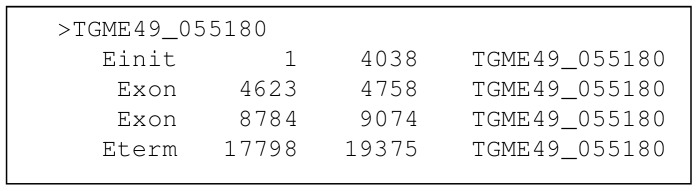
Example of exon location file. The first column is the feature name. The second and third column defines that start and end location of the exon relative to the “Einit” feature. The last column is the name of the gene sequence relative to the exon locations.

**Figure 2:**
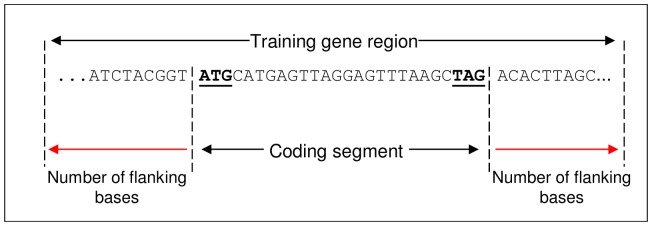
Example of flanking nucleotide bases appended to coding segment. It is required that a set number of nucleotide bases are added before and after the coding segment (CDS) sequence when assembling training genes.

All four evaluated gene finders required a training dataset. The creators of the programs SNAP, AUGUSTUS, and GlimmerHMM provide training programs that require specific input data to train hidden Markov models. The input data consists of two files: one file containing the DNA sequences of experimentally validated genes in a multi-FASTA format, and the other containing start and end exon base pair locations of these gene sequences. The format of the exon file is non-standard and is specific to each program. The exon locations are relative to the start of the gene sequence. Figure 1 shows an example format of an exon file before amendments. The gene sequence starts at position 1 and the start exon (ATG codon) starts at position 1 on the sequence and ends at 4038. As will be shown in the results, the accuracy of gene finders is governed not only by the number of validated genes in the training dataset but also by the number of nucleotide bases preceding and trailing the coding segment (CDS) sequence. Figure 2 shows an example of these flanking nucleotide bases. The exon location file must reflect the extended CDS sequences due to the flanking bases. For example, if the extension is 500 bases before and after the CDS the first exon in Figure 1 would start at 500 and end at 4538.

In-house Perl scripts were used to create the required input files for the training programs. The evaluation was performed on a chromosome per chromosome basis. A defined number of gene sequences were randomly extracted from the validation set but any target chromosome genes and genes used for testing were excluded. In addition, extracted gene sequences included a defined number of flanking bases before and after the CDS. For the evaluation the following were extracted: three sets containing 250, 500, and 1000 training genes with 250, 500, and 1000 flanking bases. In effect there were nine files containing training genes, e.g. one file contained 250 genes sequences with 250 flanking bases; another contained 250 genes with 500 flanking bases, and so on. There were also nine associated files per gene finder containing the base pair locations of the exons within these training gene sequences. For example, one of the nine files contained exon locations for 250 training genes and the locations were modified by 250 to account for the flanking nucleotides in the training gene. Each of the nine training gene files along with their associated exon location files were input one at a time into the gene finder-specific training program to create nine separate model parameters. The model parameters are essentially probability distributions used by gene finders.

### Evaluating the Accuracy of Gene Finders Using the Measures of Sensitivity and Specificity

**Figure 3:**
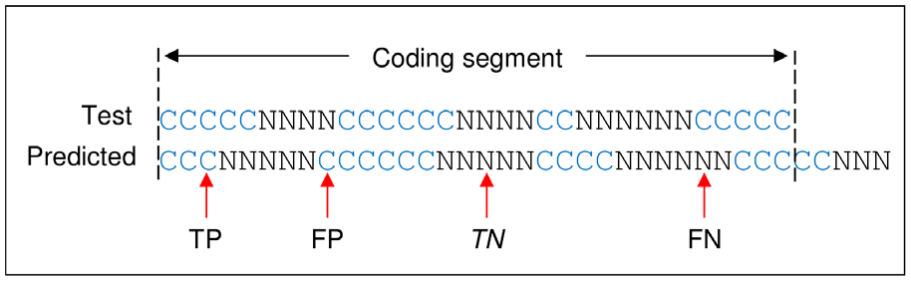
Schematic representation of gene prediction evaluation at the nucleotide level. Abbreviations: C = coding nucleotide located on exon, N = non-coding nucleotide located on intron, TP = true positive, FP = false positive, TN = true negative, and FN = false negative.

**Figure 4:**
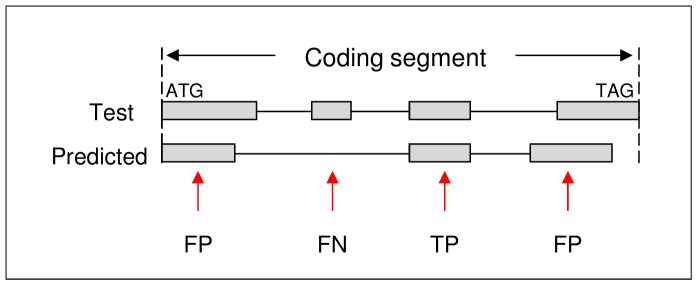
Schematic representation of gene prediction evaluation at the exon level. Exons are represented by shaded rectangles. Introns are represented by the adjoining solid lines. Abbreviations: TP = true positive, FP = false positive, and FN = false negative.

There are only four possible prediction outcomes – true positive, false positive, true negative, and false negative. Most gene finder evaluations found in the literature [8], [16], [35], [54], [55] report the accuracy of predictions using the conventional measures of sensitivity and specificity:
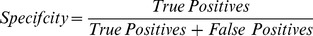



These measures were also adopted here, however, there appears to be no clear convention in the literature on how the outcome classifications should be made at each level, and in particular the exon and gene level. What follows is how the classifications were determined for the evaluation presented here. Firstly, the evaluation was performed on only one *T. gondii* chromosome. The test genes used to compare the accuracy of the predictions for the target chromosome were extracted from the validation set. These test genes represent a subset of an unknown number of genes encoded in the target chromosome. Consequently, if the predictions were evaluated with respect to the entire chromosome, only a true positive or false negative outcome could be stated with any level of certainty. For example, the outcome for a particular prediction would be classified a false positive if there is no known gene within the predicted region of the chromosome. It is possible, however, that this region could in fact encode a gene yet to be discovered experimentally. Therefore, the evaluation presented here was only performed within the CDS region of the test gene. Evaluation at the nucleotide level involved classifying each nucleotide within the CDS as coding (i.e. nucleotide located on an exon) or non-coding (i.e. nucleotide located on an intron). Figure 3 shows an example of prediction outcomes at the nucleotide level. A true positive outcome was when a predicted coding nucleotide exactly aligned with a coding nucleotide on the test gene, a false positive was when a predicted coding nucleotide aligned with a non-coding nucleotide on the test gene; and a false negative was when a predicted non-coding nucleotide aligned with a coding nucleotide. For the classification at the exon level, the exact alignment of an entire exon was a positive outcome and all other predicted exons were false positive outcomes. A false negative was when an exon in the test gene was not predicted or incorrectly predicted. Figure 4 shows an example of prediction outcomes at the exon level. For a true positive outcome at the gene level, every exon in the CDS of the predicted gene must precisely align to the respective exon in the CDS of the test gene. This is a stringent test since only one incorrect exon ensures a false positive outcome. A false negative was when a gene in the test set was not predicted or one exon incorrectly predicted. In preliminary testing of the gene finders it was shown that given the same DNA sequence, input parameters, and training data they make the same prediction each time. The gene finders in the evaluation were only executed once per test training model.

### Evaluation of Gene Finders by Aligning Predicted and Test Sequences

Predicted gene sequences derived from the nine training set files were also aligned to the test gene sequences using BLASTN. In a similar way, gene finder programs that use genome sequence comparison, such as N-SCAN [36] (a descendant from TWINSCAN 2.0, which is in turn a descendant of GenScan [26]), first perform *ab initio* gene predictions and then compare the predictions against a collection of sequences from an informant genome using BLASTN.

BLASTN is part of the BLAST suite of applications. Predicted genes that aligned with an expect value equal to 0 and with 100% coverage were of particular interest because it is an indication of how well the gene finders perform in locating the gene in a DNA sequence. The expect value, often referred to as an e-value, is a parameter that describes the number of “hits” (i.e. matches) expected due to chance when searching a database of a particular size – the lower the e-value the more “significant” the match.

### Evaluation of Gene Finders at the Protein Level

Whilst the evaluation of gene finders at the nucleotide, exon, and gene level is appropriate, evaluation at the predicted protein level is considered equally important. To reiterate, a desired goal from *ab initio* gene finders is to use the output to discover novel proteins. Incorrect predicted exons manifests in incorrect translations to amino acid residues. The question is whether the translated DNA sequence is sufficiently accurate to be used as a query to find existing proteins (homologues). The output predictions from the gene finders are in the form of exon genomic coordinates. In-house Perl scripts were used first to obtain nucleotide sequences based on the predicted exon locations from each gene finder, and then to translate into protein sequences in FASTA format. A stand-alone BLASTP was used to search a protein database using the predicted protein sequences as queries (BLASTP is also part of the BLAST suite of applications). The protein database is called “nr” and was downloaded from NCBI FTP site. The comprehensive nr database contains all non-redundant GenBank coding segment (CDS) translations, NCBI RefSeq proteins, proteins from Protein Database (PDB), UniProt, International Protein Sequence Database (PIR), and Protein Research Foundation (PRF). BLASTP aligns the amino acid residues of the predicted sequence with each nr database sequence in turn to achieve maximal levels of identity and conservation (in the case of amino acid sequences) for the purpose of assessing the degree of similarity and the possibility of homology (modified from http://www.ncbi.nlm.nih.gov/books/NBK62051/). Every alignment in which the e-value is lower than a threshold parameter is reported as a hit. In the evaluation all default BLASTP parameters were adopted and output restricted to include only: query ID, query sequence length, subject ID (e.g. ID in nr database), alignment length (same as subject sequence length), identity, and expect value. An in-house Perl script was used to parse the BLASTP output and to evaluate the consistency among the gene finders. For example, if all four gene finders significantly matched (i.e. a hit with an e-value equal to 0) with the same protein then the prediction was considered more trustworthy than a prediction made by only one gene finder. Other studies [54], [56], [57] have used BLASTP to evaluate predictions.

### Classifying the Predicted Gene Locations Relative to the Test Genes

**Figure 5:**
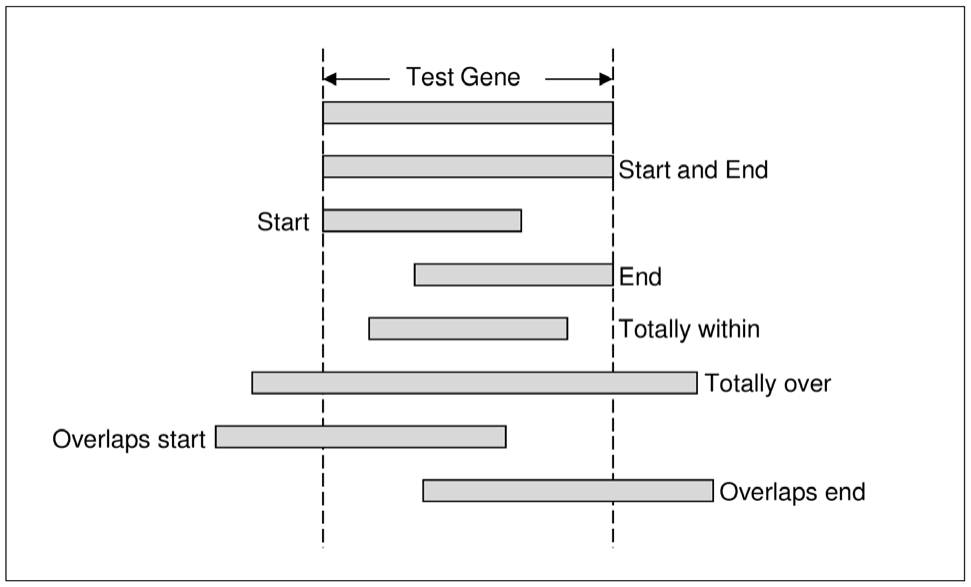
The seven classifications of the predicted gene locations relative to a test gene.

An in-house Perl script was used to evaluate the accuracy of gene finders in identifying the location of a gene within a chromosome. An incorrect start and end location of a gene manifests into an incorrect exon-intron structure. The script compared the gene locations, derived from the best predictions from each gene finder, with the start and end genomic location of the test genes. A predicted gene location can have one of seven possible locations relative to a test gene: 1) start and end location exactly the same; 2) start and end entirely within test gene; 3) start and end extend beyond test gene (i.e. test gene entirely within predicted gene); 4) start but not the end is the same; 5) end but not the start is the same; 6) end overlaps the start of test gene; and 7) start overlaps the end of the test gene. Figure 5 shows the seven classifications. Each prediction was assigned to one of the seven classifications. These classifications were used as an aid to determine how well the gene finder identified the location of genes. Another study [56] clusters predictions by genomic locations.

Similarly, a comparison of the genomic start and end locations of exon predictions with exons in the test genes was performed with an in-house Perl script. The approach used to classify the exons is similar to another study [58], except we used seven exon classifications as per Figure 5 rather than thirteen. The general aim of the approach was to identify common patterns of incorrectly predicted exons relative to the boundaries (i.e. the start and end of exons) of the test exons.

### Finding Novel Genes

Due to the inaccuracies of individual gene finders in general, it has been proposed several times in the literature [10], [55], [59] that the most judicious approach to finding genes is to combine the predictions from a pool of gene finders. There are programs such as EvidenceModeler [60], JIGSAW [61], Evigan [62], and GLEAN (http://sourceforge.net/projects/glean-gene) that combine *ab initio* gene predictions and other evidence (e.g. protein and transcript alignments) into consensus gene predictions. In this study, which is specifically to evaluate *ab initio* gene finders, the assumption is that there is no other evidence. To find potential novel genes (and ultimately novel proteins), the predicted DNA sequences from each gene finder were grouped according to similar start and end locations on the chromosome. For example, a group would contain all predicted sequences where either the start, or end, or both start and end locations of the sequences overlapped. The sequences in each group were then aligned relative to their genomic start locations. A consensus sequence was derived for each aligned group. Some predicted sequences that overlap during the grouping process can cause the consensus sequence to potentially contain more than one gene i.e. there can be multiple breaks in the consensus. For example, the aligned group may contain a length of consensus sequences followed by a stretch of sequences where no consensus can be found, and then another length of consensus sequences. Therefore, any group consensus sequence containing a break was split at the break into individual consensus sequences. It is possible, however, this action can erroneously split a group consensus that correctly represents one gene but contains a break due to poor prediction. All consensus sequences were assumed to represent one gene and were checked to ensure that they start with an “ATG” and end with either “TAG”, “TAA”, or “TGA”. The consensus sequences fulfilling this start and end requirement were used as a query to BLASTX. BLASTX is also part of the BLAST suite of applications. The BLASTX algorithm was used to compare the six-frame conceptual translation products of the consensus sequences against the protein sequences in the nr protein database. The expectation was that the regions in the consensus sequence, which represent exons, would align to known protein sequences. This expectation was based on the fact that the top BLASTX “hit” for all test genes was to relevant *T. gondii* proteins. A consensus sequence that did not align to a *Toxoplasma* protein was deemed a potential novel gene worthy of further investigation.

## Results

The data from the pathogen *T. gondii* was used to evaluate the efficacy of the *ab initio* gene finders. The purpose of the evaluation was to determine the effectiveness of *ab initio* gene finders as a whole in the discovery of novel proteins missed by laboratory techniques rather than to publish that one gene finder is necessarily better than another. The focus here is also on the performance of the gene finders and in particular possible patterns of prediction inaccuracies. No attempt is made to propose scientific findings for *T. gondii* as it is beyond the scope of the paper.

The *T. gondii* genome is approximately 63Mb in size and consists of 14 chromosomes. Gene predictions were performed on *T. gondii* (ME49 strain) chromosome VIIb downloaded from ToxoDB database at http://toxodb.org/toxo/. The length of the chromosome is 5,023,922 base pairs and reportedly contains 678 genes as per the number of entries in ToxoDB. Of the 678 gene entries, 377 are hypothetical proteins (i.e. no known homologs).

### Sensitivity and Specificity of Gene Finders

Table 2 shows the evaluation of three of the gene finders (GlimmerHMM, SNAP and AUGUSTUS) at gene, exon, and nucleotide level. GeneMark_hmm was excluded from the sensitivity and specificity measures because it provides no facility to create user-defined training models. GeneMark_hmm provides instead a readymade training set for *T. gondii*. The test genes, and therefore genes on the target chromosome VIIb, were excluded from the training genes in this evaluation stage. There were three main points inferred from this evaluation test. The first point was that the gene finders have extremely low accuracy in precisely determining the exon-intron structure of gene. For example, the highest exon sensitivity (SN) and specificity (SP) was for AUGUSTUS with 0.52 and 0.56 respectively, followed by 0.47 and 0.37 for SNAP, and then the least accurate 0.27 and 0.31 for GlimmerHMM. The second point is that the accuracy of gene predictions improves as the number of training genes increases. This result is as expected, however, an inherent bias when using training datasets is that algorithms will find genes most similar to those in the training data [41]. Determining the optimal number of training genes is a challenge. Table 3, for example, clearly shows that using all validated genes (3,432 including the test genes) in the training model gives the best results. It is impossible nevertheless to ascertain whether only genes similar to training genes are found because the complete number of true genes on the chromosome is unknown, i.e. it is not possible to check for overtraining. Therefore, we propose that it is currently an art to achieve an optimal balance between accuracy and overtraining. In addition to the number of genes in the training set the prediction accuracy also depends on the variety of genes in the training set. A training set in effect represents an “average gene” so it can be expected that some genes in the genome will not be predicted or will be incorrectly predicted because they greatly differ in structure or compositional biases to the so-called average gene [34]. The third point inferred from Table 2, and supported by Table 4, is that the number of nucleotides before and after the CDS impacts accuracy but in a different manner for each gene finder. For example, GlimmerHMM tends to be more accurate with more flanking nucleotides, whereas SNAP tends to be the opposite. The utilisation of these intricacies of different flanking requirements for each gene finder is recommended in the deployment stage of the gene finder. Table 3 also shows that using a HMM model trained on humans substantially reduces the prediction accuracy and these results further highlight the need to use only models trained for the target organism.

**Table 2:** Evaluation of gene finders at the gene, exon, and nucleotide level (with 250, 500, and 1000 training genes).

GeneFinder	# of flankingbases	Accuracy level																	
		GENE						EXON						NUCLEOTIDE					
		Number of training genes$$																	
		250		500		1000		250		500		1000		250		500		1000	
		SN	SP	SN	SP	SN	SP	SN	SP	SN	SP	SN	SP	SN	SP	SN	SP	SN	SP
**gl**	**250**	0.11	0.11	0.13	0.13	0.12	0.11	0.24	0.26	0.27	0.31	0.25	0.27	0.52	0.49	0.56	0.56	0.52	0.48
	**500**	0.11	0.11	0.13	0.12	0.13	0.12	0.23	0.25	0.26	0.29	0.26	0.29	0.51	0.48	0.55	0.53	0.54	0.50
	**1000**	0.11	0.11	0.14	0.14	0.14	0.14	0.23	0.26	0.26	0.30	0.27	0.29	0.51	0.47	0.56	0.54	0.57	0.54
**snap**	**250**	0.11	0.07	0.15	0.10	0.15	0.10	0.40	0.27	0.42	0.30	0.47	0.37	0.44	0.37	0.45	0.38	0.46	0.37
	**500**	0.14	0.08	0.15	0.10	0.16	0.11	0.40	0.28	0.40	0.28	0.42	0.31	0.44	0.36	0.44	0.36	0.46	0.36
	**1000**	0.13	0.08	0.14	0.10	0.15	0.10	0.37	0.26	0.32	0.23	0.37	0.27	0.43	0.35	0.42	0.32	0.41	0.32
**aug**	N/A	0.24	0.28	0.27	0.31	0.30	0.35	0.44	0.52	0.47	0.52	0.52	0.56	0.78	0.72	0.80	0.76	0.81	0.78

$$ Test genes (299 in total) are excluded from the training genes.

The values underlined indicate the highest accuracy in each accuracy level for each gene finder.

Abbreviations:

SN = sensitivity, SP = specificity.

gl = GlimmerHMM; aug = AUGUSTUS.

N/A = not applicable – the AUGUSTUS training program does not give the option to control the number of bases that precede and follow the coding segment (CDS) sequences of the training genes.

**Table 3:** Evaluation of gene finders with various training genes.

GeneFinder	Traininggenes##	Accuracy level								
		GENE		EXON		NUCLEOTDE				
		SN	SP	SN	SP	SN	SP	Predicted	Matched$$	Duplicate++
**GlimmerHMM**	All validated genes excepttest genes	0.16	0.15	0.27	0.30	0.54	0.50	684	269 (47)	52
	All validated genes includingtest gene	0.20	0.20	0.33	0.35	0.61	0.55	710	273 (64)	47
	Using a trained model fromprogram creator	Not available for *Toxoplasma gondii*								
	Using a model trained onhuman genes	0.02	0.01	0.04	0.05	0.23	0.14	1129	247 (5)	131
**SNAP**	All validated genes excepttest genes	0.18	0.12	0.44	0.33	0.46	0.35	889	277 (53)	172
	All validated genes includingtest genes	0.18	0.12			0.46	0.35	895	279 (54)	170
	Using a trained model fromprogram creator	Not available for *Toxoplasma gondii*								
	Using a model trained onhuman genes	0.09	0.04	0.06	0.09	0.16	0.11	1759	267 (25)	315
**AUGUSTUS**	All validated genes excepttest genes	0.33	0.38	0.54	0.57	0.81	0.78	510	261 (99)	2
	All validated genesincluding test genes	0.37	0.42	0.57	0.59	0.82	0.79	514	265 (111)	2
	Using a trained modelfrom program creator	0.36	0.42	0.57	0.56	0.78	0.84	470	256 (108)	0
	Using a model trained onhuman genes	0.12	0.09	0.19	0.19	0.34	0.25	114	282 (37)	150
**GeneMark_hmm**	Using a trained modelfrom program creator	0.06	0.07	0.15	0.13	0.43	0.37	580	240 (19)	49
**GeneMark_hmm ES**	Using a self-training procedure.i.e. no training genes required	0.08	0.09	0.23	0.19	0.56	0.44	630	248 (25)	45

## The types of training genes used in the training model. The number of validated genes  =  3,432 (includes test genes) and the number of test genes  =  299.

$$ Number of predicted genes that align entirely or partly with the test genes and meet the criteria E-value  =  0 and 100% coverage – a value in brackets is the number of predicted genes that are exactly the same as the test genes i.e. the start and end genomic coordinates of each exon is the same as each test gene exon.

++ Number of predicted genes that align to the same test gene i.e. the predicted gene is only a part of the entire test gene and there can be one or more predictions per test gene.

On closer examination of Table 2 and Table 3 the specificity of SNAP is significantly lower than sensitivity. This is because the specificity calculation is distorted by partial predictions. For example, the predictions made by SNAP align to only a part of the test gene such that there tends to be more than one prediction per test gene and some predictions overlap. The accuracy shown in Table 3 for GeneMark_hmm is clearly much lower than the other gene finders. Surprisingly the self-training version of GeneMark, called GeneMark_hmm ES [63], is more accurate. However, the history and the content of the *T. gondii* trained model used in the evaluation of the supervised GeneMark_hmm are unknown and hence it would be naive, and unfair to the creators, to conclude that GeneMark_hmm is the least accurate gene finder.

**Table 4:** Number of matching predicted genes with 299 test genes using BLASTN (with 250, 500, and 1000 training genes).

Gene Finder	# of flanking bases	Number of training genes								
		250			500			1000		
		Predicted	Matched$$	Duplicate++	Predicted	Matched$$	Duplicate++	Predicted	Matched$$	Duplicate++
**gl**	250	575	255 (34)	43	627	261 (40)	44	666	260 (35)	58
	500	579	254 (33)	45	631	263 (38)	44	668	263 (34)	48
	1000	594	256 (33)	47	640	259 (43)	44	659	263 (34)	45
**snap**	250	882	273 (34)	192	891	271 (45)	186	892	276 (46)	169
	500	880	266 (40)	193	851	265 (44)	187	862	270 (48)	172
	1000	824	256 (39)	184	829	255 (43)	189	838	262 (45)	182
**aug**	N/A	485	248 (72)	5	506	256 (82)	6	508	258 (90)	2

Abbreviations:

gl = GlimmerHMM; aug = AUGUSTUS.

N/A = not applicable – the AUGUSTUS training program does not give the option to control the number of bases that precede and follow the coding segment (CDS) sequence of the training genes.

$$ Number of predicted genes that align entirely or partly with the test genes and meet the criteria E-value  =  0 and 100% coverage – a value in brackets is the number of predicted genes that are exactly the same as the test genes i.e. each exon genomic coordinate is the same.

++ Number of predicted genes that align to the same test gene i.e. the predicted gene is only a part of the entire test gene and there can be one or more predictions per test gene.

The values underlined indicate the highest number of matches for each gene finder.

### How Well Predicted Sequences Align to Test Sequences

Table 4 shows the results from aligning the predicted gene sequences with the test gene sequences (299 in total) using BLASTN. As expected, the results supported the findings in Table 2 and Table 3 i.e. there is an increase in the number of matches with more genes in the training set. The number of predicted genes also increases as the number of training genes increases. For example, GlimmerHMM made 594 predictions with 250 training genes and 659 predictions with 1000 training genes. The increase in the number of predictions (65 in this case) is most likely because of the increase in variety of structure and compositional biases brought about by the increase in training genes. In other words, the additional predictions did not conform to the “average gene” of the 250 training set.

Despite the low accuracy of *ab initio* gene finders for precise genomic annotation, the results in Table 4 are still encouraging with respect to locating the genomic location of the gene within the chromosome, even though the predicted exons may be incorrect. GlimmerHMM, for instance, only precisely predicted 34 genes where all exon locations were correct out of a possible 299. The predicted nucleotide sequence, however, is of sufficient accuracy to significantly align with 263 test genes.

The bottleneck to *in silico* discovery of proteins is the inaccurate prediction of exon location within the DNA sequence i.e. the DNA predictions translated to amino acids are imprecise since their predicted exons are imprecise. Hence one prediction from one gene finder is too uninformative to propose it is a novel protein. However, if several gene finders all predict a similar gene region within the genome then it is more likely that the genome encodes a gene. The likelihood that it is a true novel gene increases when other gene finder predictions in agreement can be matched to experimentally validated genes.

### Evaluation at Protein Level

Table 5 shows the results after translating the concatenated DNA sequences of the predicted exons into amino acids and using them as a query in BLASTP to find matching proteins in the protein database (nr). As an example of interpretation, AUGUSTUS predicted 514 genes, of which 509 predictions (after translation) significantly matched an existing protein and 5 did not. Unmatched queries are most likely due to the inaccuracy of predicted exons. It is nevertheless possible that some unmatched queries are novel proteins.

**Table 5:** Protein homology search on translated gene finder predictions.

Gene Finder	Gene predictions	Homology found$$	Homology not found
AUGUSTUS	514	509	5
GeneMark.hmm	580	481	99
SNAP	895	734	161
GlimmerHMM	710	657	53

$$ Includes duplicate proteins. Duplicate proteins are when several gene predictions match to the same protein.

A predicted protein sequence of a gene finder can match more than one protein in the nr database. For example, in the case for AUGUSTUS there were 1,194 significant (e-value  =  0) protein matches from 514 predicted protein sequences. There are two possible reasons for this: 1) the same protein sequence has been incorrectly added multiple times in nr under different IDs, or 2) it is a different form of the same protein i.e. alternative splicing has occurred.

If gene finders can successfully predict a certain percentage of known proteins then we propose that it is reasonable to assume a similar percentage of predictions with no homologous matches are novel proteins. This assumption has more weight if all gene finders predict a similar genomic location within the chromosome for the same gene prediction. Table 6 shows the number of identical predicted proteins found in the protein database (nr) per number of gene finders. For example, 923 unique predicted proteins were identified by all four gene finders. There were 1,603 unique predicted proteins found in the protein database (nr) from a combined number of 5,323 hits. Encouragingly the majority of these unique proteins were found by all four gene finders and consequently provide sound evidence that these translated DNA sequence are of sufficient accuracy to be used as queries to find existing proteins (homologues). Proteins found by only one or two gene finders are questionable predictions.

**Table 6:** Identical proteins found in protein database per number of gene finders.

No. of proteins	No. of Gene Finders	Gene Finders
923	4	AUGUSTUS, Glimmer, GeneMark, SNAP
257	3	AUGUSTUS, Glimmer, GeneMark
84	3	Glimmer, GeneMark, SNAP
25	3	AUGUSTUS, Glimmer, SNAP
8	3	AUGUSTUS, GeneMark, SNAP
57	2	Glimmer, GeneMark
43	2	AUGUSTUS, Glimmer
25	2	Glimmer, SNAP
14	2	AUGUSTUS, SNAP
8	2	AUGUSTUS, GeneMark
8	2	GeneMark, SNAP
23	1	SNAP
27	1	AUGUSTUS
34	1	GeneMark
67	1	Glimmer
**1603 = ** Total number of unique proteins		

### Prediction Statistics

Table 7 shows some statistics for the predicted and test genes. The aim here was to identify similarities or differences between the gene finders. For example, AUGUSTUS has the largest average and longest distance between gene predictions. Predictions for all gene finders are made throughout the chromosome and are evenly distributed as shown by the predictions located in four chromosome partitions. AUGUSTUS also differs from the other gene finders by making no predictions in the first 54,055 nucleotides (as is the case for SNAP) and the last 21,392 nucleotides of the chromosome. SNAP did not predict any single exon genes. In contrast, GlimmerHMM predicted substantially more single exon genes than any other gene finder.

**Table 7:** Statistics for predicted and test genes.

Statistics for …	AUGUSTUS	GlimmerHMM	SNAP	GeneMark_hmm	Test genes
Number of genes	514	710	895	580	299
**Gene Length**					
Shortest	270	201	399	303	298
Longest	44325	37271	22713	45369	47133
Average	5733	5677	4679	7979	5388
Range	44055	37070	22314	45066	46835
Number of genes containing an N	10	17	22	19	5
**Distance between genes**					
Shortest	29	0	0	104	52
Longest	31658	8549	21815	4677	106560
Average	3894	1398	3112	664	11081
Range	31629	8549	21813	4573	106508
Percentage of overlaps	0	0.4	26.9	0	0
**Chromosome gene region**					
Length of chromosome	5023922	5023922	5023922	5023922	5023922
Start of first gene	54055	635	54055	7447	78150
End of last gene	5002530	5020498	5023141	5020134	5002376
Range	4948475	5019863	4969086	5012687	4924226
Distance to start of chromosome	54055	635	50455	7447	78150
Distance to end of chromosome	21392	3424	781	3788	21546
**Percentage of genes located in:** $$					
Partition 1	26.1	26.3	25.1	27.1	26.4
Partition 2	24.7	23.3	25.6	24.5	25.7
Partition 3	24.5	25.1	24.5	24.1	21.4
Partition 4	24.7	25.2	24.8	24.3	26.4
**Exons**					
Total number	3357	3334	4746	4172	2013
Shortest exon	3	5	5	7	3
Longest exon	9981	9981	9977	9985	9981
Average length	403	448	364	380	350
Average number per gene	7	5	6	8	7
Highest number per gene	46	31	29	47	47
Lowest number per gene	1	1	2	1	1
Number of single exons	67	123	0	63	39
**Introns**					
Total number	2844	2624	3851	3592	1714
Shortest intron	43	4	4	23	51
Longest intron	5834	5707	6734	9961	3560
Average length	560	968	640	848	530
Average number per gene	6	4	5	7	6
Highest number per gene	45	30	28	46	46
Lowest number per gene	1	1	1	1	1

$$ The target chromosome was divided into four equal parts (partitions 1 to 4). The genomic location of each gene prediction determined the relevant partition allocation.

A study [56] using three gene finders (GeneZilla [34], Twinscan [31], and GlimmerHMM) to predict genes in *T. gondii* chromosomes showed that the length of the translated gene sequences for GlimmerHMM (1,077 residues average length) were on average much longer than GeneZilla (681 residues) and TwinScan (614 residues) translated predictions. No average genes lengths were reported but the latter implies that GlimmerHMM predicts much longer genes or predicts more exons or longer exons than the other two gene finders. Our study suggests longer exons as GlimmerHMM predicted gene sequences with an average length of 5,677 that was less than the predicted lengths of AUGUSTUS and GeneMark_hmm but greater than SNAP. The average number of exons per gene (5 exons) for GlimmerHMM was the smallest of all the evaluated gene finders but the average length of the exons (448 nucleotides) was the largest.

### Classification of Predicted Gene Locations Relative to the Test Genes

Table 8 shows a comparison of the genomic start and end locations of the gene predictions with the test genes. The start location in this instance is the start of the initial codon (ATG) and the end location is the end of the stop codon (TAG, TAA, or TGA). AUGUSTUS clearly performed the best in locating the start and end of genes precisely with 152 out of 299 but failed to identify 26 of the test genes. AUGUSTUS was evaluated along with GeneMark_hmm, GeneZilla, GeneID, and GenScan in the human encode genome annotation assessment project (EGASP) and was shown to consistently find the start and end of genes better than the other evaluated *ab initio* gene finders [55]. Conversely GeneMark_hmm precisely identified only 31 but identified (by partial overlapping) all 299 test genes. GeneMark_hmm overlaps with more test genes because the average length of its predicted genes is considerably greater than the predicted genes of the other evaluated gene finders (see Table 7 for statistics on predicted genes). GeneMark’s prediction length is too long and this is supported by the fact that 116 predicted locations are classified as “totally over” i.e. the test gene is located entirely within the predicted gene. SNAP prediction lengths, on the other hand, are too short since 90 are classified as “totally within” i.e. the predicted gene is located entirely within the test gene. This finding is also supported by the fact that 197 out of 895 predictions constitute partial predictions and the average prediction length (Table 7) is shorter than other gene finder average lengths. Partial predictions are when only part of an entire gene is predicted such that there can be more than one prediction to the same test gene by the same gene finder. SNAP makes the most partial predictions of which 26.9% overlap (see Table 7).

**Table 8:** Comparison of genomic start and end locations of gene predictions with 299 test genes.

Classification**	gm	aug	gl	snap	all	aug:gl:snap	aug:gl	aug:snap	aug:gm	gl:snap
Start and End	31	152	93	102	89	112	127	125	116	109
Start	55	47	76	69	70	64	60	65	58	68
End	57	57	85	82	104	92	81	84	82	98
Totally Within	27	4	21	90	75	47	29	21	39	51
Totally Over	116	7	27	27	3	3	4	4	6	7
Overlaps Start	42	6	29	49	7	5	7	6	10	13
Overlaps End	40	5	27	61	7	7	7	6	10	11
**Summary (Number of …)**										
Predictions	580	514	710	895	666	624	594	584	585	730
Test genes identified	299	273	297	283	267	271	273	271	268	281
Test genes not identified	0	26	2	16	32	28	26	28	31	18
Matches with test genes(includes partial predictions)	368	278	358	480	355	330	315	311	321	357
Partial predictions++	69	5	61	197	88	59	42	40	53	76
Non-matches$$	212	236	352	415	311	294	279	273	264	373

Abbreviations:

gm = GeneMark_hmm, aug = AUGUSTUS, gl = GlimmerHMM.

** See Figure 5 for explanation on classifications.

++ Number of predicted genes that predict part of an entire gene such that there can be more than one prediction to the same test gene.

$$ Number of predictions that did not overlap the test genes in any way.

All gene finders precisely locate the end of a gene slightly more often than the start of a gene. This is expected due to the fact that it is difficult to distinguish a functional start codon (ATG) from other ATG triplets in the DNA sequence that code for the amino acid methionine. Stop codons on the other hand normally do not code for any amino acids. Table 8 also shows the location classifications when using a consensus of the gene finder predictions. For example, consensus sequences constructed from all four gene finders precisely define the start and end of 89 test genes. Different combinations of gene finders were tested to achieve the optimal combination. The combination of AUGUSTUS and Glimmer marginally achieved the best result with 268 gene boundaries precisely identified (start and end  =  127, start  =  60, end  =  81) and 273 test genes identified.

A question that arose was why the gene finders, and in particular AUGUSTUS, failed to identify some of the test genes. The test genes not found were compared to see if there was any similarity or pattern to these genes. The results are shown in Table 9. AUGUSTUS failed to identify 26 test genes of which 61.5% were genes from the reverse strand, 50% were single exon genes, and the average length of the test genes not found was 67.5% less than the average length of all test genes. In addition, 70% of the single exon genes were from the reverse strand. The two test genes not identified by GlimmerHMM were single exons genes both from the reverse strand. SNAP failed to identify 16 test genes of which 75% were from the reverse strand, 56% were single exon genes, and the average length was 65.1% less than the average length of all test genes. Table 10 shows the number of test genes not found that are in common with the evaluated gene finders. It is proposed from these findings that the gene finders, in general, have greater difficulty in identifying single exon genes or shorter than average length genes that are located on reverse strands.

**Table 9:** Comparison of test genes not identified by gene finders.

Statistics for …	AUGUSTUS	GlimmerHMM	SNAP	Test genes
Test genes not identified	26	2	16	299
Reverse strand	16	2	12	153
Consecutive groups++	3	0	2	–
Highest consecutive number$$	4	0	3	–
Number containingan N	0	0	0	5
**Gene Length**				
Average	1861	573	1996	5733
Shortest	342	492	342	298
Longest	7332	654	7332	47133
**Distance to next test gene**				
Shortest	52	248	52	52
Longest	69635	7237	69635	106560
Average	11127	2271	10515	11081
**Exons**				
Shortest exon	14	492	14	3
Longest exon	4149	654	1827	9981
Average length	214	573	119	350
Average number per gene	4	1	5	7
Highest number per gene	15	1	15	47
Lowest number per gene	1	1	1	1
Number of singleexons	13	2	9	39
**Introns**				
Shortest intron	51	0	51	51
Longest intron	1074	0	1074	3560
Average length	68	0	43	530
Average number per gene	3	0	4	6
Highest number per gene	14	0	14	46
Lowest number per gene	1	0	1	1

++ Number of groups of test genes not found in which the test genes are located consecutively along the chromosome.

$$ The highest number of test genes in a consecutive group.

**Table 10:** Commonality of test genes not identified by gene finders.

Commonality	Number of genesnot found	Single exon gene	Reverse strand	% less thanaverage length**
AUGUSTUS, Glimmer, SNAP	1	1	1	89
AUGUSTUS, SNAP	13	7	9	64
AUGUSTUS, Glimmer	1	1	1	91

** The percentage less than the average length of all the test genes.

Table 11 shows the comparison of the genomic start and end locations of exon predictions with exons in the test genes. The values in the table are percentages of the number of exons falling into one of the seven classifications. For exons that were classified, there were more exons in classification one (start and end boundary correct) than any other classification. More start boundaries were correctly identified, which suggests that all gene finders have a greater difficulty in predicting end boundaries of exons. Many test exons were not overlapped in any way. For example, 40% of the test exons were missed by GeneMark_hmm. It is tempting to assume here that SNAP performed the best by only missing 9% of the test exons, but some of SNAP’s exon predictions are duplicates.

In a similar exon classification study [58], four *ab initio* gene finders (AUGUSTUS, Genezilla [34], GlimmerHMM, and SNAP) and two comparative genomics gene finders (GenomeScan [30] and Twinscan [31]) were tested on human DNA. Each evaluated gene finder was trained on a different dataset. With reference to the exon classification in Figure 5 and Table 11, the findings of the latter study were mostly in agreement with our results: 1) the gene finders predicted more class 1 (start and end boundary correct) than any other class (GeneMark and GlimmerHMM are the exceptions in our study); 2) the second largest class was for predicted exons that did not overlap the test exons in any way; 3) the third largest class was for exons that correctly matched either the start boundary (class 2) or end boundary (class 3) – in our study, there were more predictions that overlapped either the start boundary (class 6) or end boundary (class 7) than class 3; 4) no exons were predicted such that their end boundary matched the test start boundary or their start boundary matched the test end boundary; 5) the predicted start boundary of an initial exon of a multi-exon gene tended to occur after the test start when the predicted end boundary was correct i.e. the gene finders tended to incorrectly predict shorter initial exons; 6) the predicted end boundary of a terminal exon tended to occur after the test end when the predicted start boundary was correct i.e. gene finders were more likely to predict exons extending beyond the stop codon; 7) no gene finder predicted a single exon gene that started correctly and ended before the stop codon; and 8) incorrectly predicted exons that did not overlap the test exon in any way, occurred more often after the test exon than before.

**Table 11:** Comparison of genomic start and end locations of exon predictions with exons in test genes (values are percentages).

Classification	GeneMark_hmm	GeneMark_hmm ES	AUGUSTUS	GlimmerHMM	SNAP
1. Start and End	16	23	57	33	44
2. Start	13	15	7	12	19
3. End	1	3	2	1	2
4. Totally Within	3	3	1	2	3
5. Totally Over	9	9	2	5	6
6. Overlaps Start	11	8	5	7	9
7. Overlaps End	7	6	6	6	8
Number of exons not classified (no overlap)	40	33	20	34	9

### Potential Novel Genes

**Figure 6:**
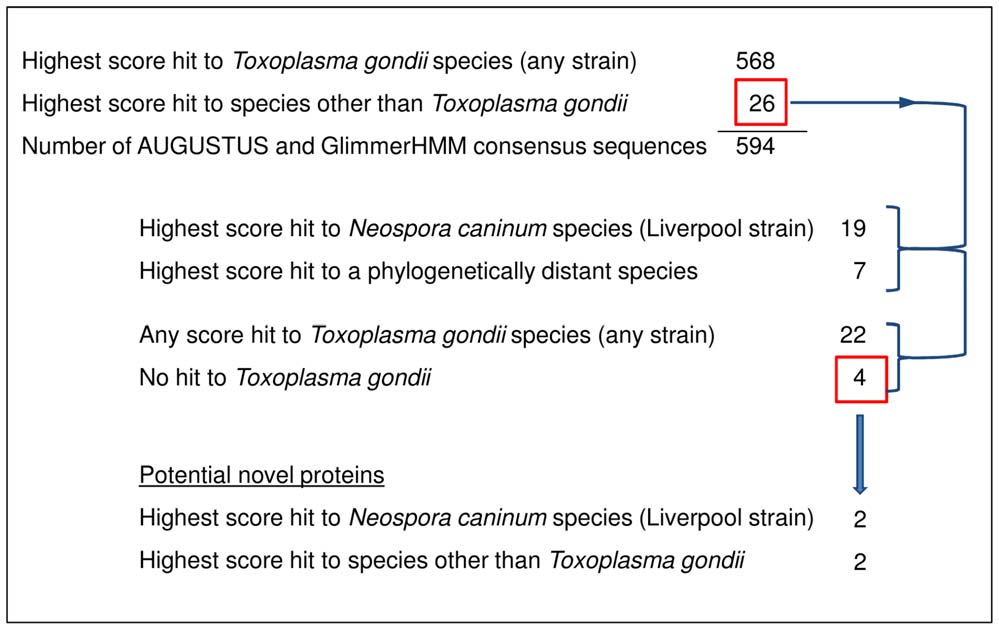
Number of BLASTX hits using DNA consensus sequences from AUGUSTUS and GlimmerHMM predictions. The figure shows the BLASTX hits when using the consensus of predicted sequences from AUGUSTUS and GlimmerHMM as queries in an attempt to find novel *Toxoplasma gondii* proteins. These consensus sequences were derived from aligning predicted DNA sequences based on overlapping genomic locations (see text for details).

Figure 6 shows the results for finding potential novel genes on chromosome VIIb using the consensus of predicted sequences from AUGUSTUS and GlimmerHMM. These consensus sequences were derived from aligned grouped sequences based on overlapping genomic locations of the predicted DNA sequences. BLASTX was used to determine if any part of the consensus sequences aligned with existing *T. gondii* proteins. There were a total of 594 consensus sequences where each sequence represented one gene. The highest BLASTX score hit for 568 consensus sequences was to the *T. gondii* species. Therefore, 26 out of the 594 consensus sequences had the highest scoring hit to a species other than *Toxoplasma*. The highest score hit for 19 of these 26 was to a phylogenetically similar species called *Neospora caninum.* When considering any score, irrespective of how low, 22 of the 26 had a hit to *T gondii.* So in summary, four sequences out of 594 did not have a hit of any kind to a known *T. gondii* protein. We propose that these four consensus sequences are potential novel proteins and are worthy of further investigation. Moreover, these results indicate that candidate novel proteins can be identified using gene finder consensus.

There are 678 genes located on the chromosome as per the number of entries in ToxoDB. We estimate that the number of genes is between 553 and 653. This estimation is based on the number of predicted sequences of each gene finder, the number of partial predictions of the same gene by the same gene finder, and the number of test genes identified or not identified. Considering the small number of consensus sequences that did not have a hit to a known *T. gondii* protein, we propose that the vast majority of genes located on chromosome VIIb have been found. These results are simply an illustration to show that it is feasible to identify the approximate location of every gene in a genome by using a pool of *ab initio* gene finders.

## Discussion

There is perception in the scientific community that *ab initio* gene finding is diminishing in importance as RNA-seq grows in importance. It is argued here that there is still a need for research into finding genes using DNA sequence alone as wet laboratory experiments currently cannot capture all mRNA. The primary purpose of this paper was to ascertain if existing *ab initio* gene finders had sufficient accuracy to capture all mRNA. Four gene finders (AUGUSTUS, GeneMark.hmm, GlimmerHMM, and SNAP) were selected as representatives of high-throughput *ab initio* gene finders to evaluate their effectiveness in discovering proteins encoded in eukaryotic pathogen genomes. The main findings of the evaluation showed that all four gene finders had low accuracies of prediction when using the conventional measures of sensitivity and specificity. Table 12 shows the accuracy of predictions from previous studies using the same gene finders. The table also shows for comparison the best accuracy results from the work conducted for this paper. Realistically the prediction accuracies can only be compared if the gene finders were trained on the same genes and the predictions were to the same target organism. Also in previous studies [9], [34], [35], [55], [58], [63], although the same standard equation for sensitivity and specificity was adopted, the precise method of how the prediction outcomes were interpreted is not clear. For example, what constitutes a false negative exon is open to various interpretations. Is it an exon in the test gene that was not predicted, or is it an exon in the test gene that was either partly predicted or not at all? Similarly, the specific criteria used to classify a true positive gene or exon may also be open to various interpretations. For example, a true positive exon could either be one that partly overlaps a test exon, or overlaps above a certain threshold, or overlaps a test exon precisely. The accuracy values in the table do, however, provide an indication of the general trend of gene finder performance irrespective of training data, organism, and evaluation method. The general trend in previous studies [9], [34], [35], [55], [58], [63] is that prediction accuracy increases as per the following gene finder order: GeneMark_hmm, SNAP, GlimmerHMM, and AUGUSTUS. The findings presented here support this general trend of accuracy performance.

**Table 12:** Accuracy of predictions from previous studies (grouped according to target organism).

Gene finder	Gene		Exon		Nucleotide		Organism	Publication
	SN	SP	SN	SP	SN	SP		
SNAP	0.54	0.47	0.83	0.81	0.97	0.95	*Arabidopsis thaliana*	SNAP creator [9]
GlimmerHMM	33%		0.71	0.79	96%		*Arabidopsis thaliana*	gl creator [34]
GlimmerHMM	21%		0.36	0.49	91%		*Aspergillus fumigatus*	gl creator [34]
SNAP	0.51	0.38	0.79	0.67	0.94	0.87	*Drosophila melanogaster*	SNAP creator [9]
AUGUSTUS	0.51	0.32	0.77	0.68	0.92	0.89	*Drosophila melanogaster*	SNAP creator [9]
AUGUSTUS	0.68	0.38	0.85	0.86	0.98	0.93	*Drosophila melanogaster*	aug creator [35]
AUGUSTUS	–	–	–	–	0.92	0.88	*Drosophila melanogaster*	gm creator [63]
SNAP	–	–	–	–	0.94	0.86	*Drosophila melanogaster*	gm creator [63]
GeneMark_hmm					0.93	0.88	*Drosophila melanogaster*	gm creator [63]
AUGUSTUS	0.47	0.51	0.71	0.79	–	–	*Drosophila melanogaster*	Independent [57]
AUGUSTUS	0.48	0.47	0.80	0.81	0.93	0.90	*Homo sapiens*	aug creator [35]
AUGUSTUS	0.24	0.17	0.52	0.63	0.78	0.75	*Homo sapiens*	Independent [55]
AUGUSTUS	–	–	0.64	0.63	0.81	0.78	*Homo sapiens*	Independent [58]
GeneMark_hmm	0.17	0.08	0.48	0.47	0.76	0.62	*Homo sapiens*	Independent [55]
GlimmerHMM	–	–	0.69	0.63	0.89	0.79	*Homo sapiens*	Independent [58]
SNAP	–	–	0.40	0.36	0.72	0.71	*Homo sapiens*	Independent [58]
AUGUSTUS	0.37	0.38	0.57	0.59	0.82	0.79	*Toxoplasma gondii*	This paper
GeneMark _hmm	0.06	0.07	0.15	0.13	0.43	0.37	*Toxoplasma gondii*	This paper
GlimmerHMM	0.20	0.20	0.33	0.35	0.61	0.55	*Toxoplasma gondii*	This paper
SNAP	0.18	0.12	0.44	0.33	0.46	0.35	*Toxoplasma gondii*	This paper

% indicates the percentage of genes and nucleotides predicted exactly. There were no SN or SP values for GlimmerHMM at the gene and nucleotide level.

– No values available.

The following is a summary of patterns of inaccuracy specific to the gene finder: SNAP did not predict single exon genes and tended to make short partial predictions of the same gene – some of these predictions were duplicates and some partially overlapped; GlimmerHMM predicted more single exon genes, less exons per gene, and longer exons than any other gene finder; GeneMark_hmm had a tendency to incorrectly predict the initial or terminal exon such that the prediction length was longer than the test gene. No previous study could be found in the literature that was specific to the evaluated gene finders when used on apicomplexan genomes. There is one study [56] that used three gene finders (GeneZilla [34], Twinscan [31], and GlimmerHMM) to identify genes in the ME49 strain of *T. gondii*. The study reported that GlimmerHMM was the least accurate of the three and the overall false negative rate for all three was about 31–42% (i.e.proteins that were found experimentally were missed by one or more of the gene finders) [56]. Several studies [56], [64], [65], [66] have used transcriptomics and proteomic techniques to evaluate current genome annotations and improve proteome datasets in key apicomplexan pathogens.

One of the primary aims of the paper was to identify possible patterns of prediction inaccuracies for the evaluated gene finders as a whole irrespective of the target pathogen. From the findings (see results section for details) the gene finders are inferred to have the following characteristics when used on any target pathogen: they predict exons of insufficient accuracy to find novel proteins without the support of experimental evidence (in agreement with [8], [56], [67]); locate the end of a gene precisely more often than the start of a gene (in agreement with [56]); improve in prediction accuracy as the number of training genes increase; increase the number of predictions as the number of training genes increase; show a tendency not to predict single exon genes or shorter than average length genes that are located on reverse strands; exhibit a change in accuracy as the number of nucleotides before and after the CDS is varied but in a different manner for each gene finder; perform better when using a model trained on a target rather than a foreign organism (in agreement with [9]); show greater difficulty in predicting initial and terminal exons compared to internal exons (in agreement with [55], [56]); have a tendency, when incorrectly predicting an initial or terminal exon, to predict the initial exon shorter and the terminal exon longer than the true exon (in agreement with [58]); show greater difficulty in predicting end boundary of exon compared to start boundary.(the opposite is reported in [58]); predict more false positive exons when pathogen has many introns per gene or has long intergenic regions (in agreement with [55]); and predict more false negative exons when pathogen has many exons per gene (in agreement with [55]).

The low prediction accuracies suggest that existing *ab initio* gene finders have insufficient accuracy to instill confidence that novel proteins can be found. For example, in an ideal scenario gene finders would precisely predict the start and end location of each exon on every validated test gene. In such a scenario, one could assume that exons from predictions that had no homologous genes were correct and encoded for novel proteins. Currently, a great proportion of the exons in the test genes are incorrectly predicted. Hence there can be no confidence in the predicted exons not located on test genes. One of the goals of EGASP was to assess whether gene finders can replace manual annotations. The conclusion was that no gene finder can deliver perfect predictions even when all computational methods – *ab initio*, evidence based and genome sequence comparison – were employed (including AUGUSTUS and GeneMark_hmm). Although the evaluated gene finders had an overall accuracy of more than 80% in identifying exons correctly, only about 60% of the annotated protein-coding transcripts were predicted [55]. The current best solution for finding novel proteins when experimental evidence is unavailable is to combine the predictions from a pool of *ab initio* gene finders as proposed in the literature [55], [59], [60], [61], [62].

Despite the overall low accuracy, *ab initio* gene finders can be used to locate the approximate location of genes in a genome. We demonstrated that by using a pool of gene finders the start and end location of every possible gene can theoretically be identified. We also suggest that using nucleotide sequences defined by these start and end locations as separate input sequences to a gene finder will increase overall prediction accuracy, rather than using an entire chromosome as input.

We acknowledge that some equally appropriate high-throughput *ab initio* gene finders may have been unintentionally missed during our selection process. At the onset, there were an overwhelming number of gene finders in the literature to choose from. In one sense, the post-genomic era has experienced a gold rush and it is equally difficult for researchers to find the ‘gold’ standard gene finders among so many. Without actually running the programs it is problematic to determine their efficacy. Occasionally, program evaluation papers can be found but become quickly out-dated as new gene finders emerge. Often the methods behind the programs are *hidden* from the user, leading to uncertainties about their confidence, accuracy and information content [44]. Conversely, the methods are written in the literature but are too technically and computationally sophisticated for a biologist to fully understand. It is of course the expertise of a biologist that in effect converts a program output into scientific findings worthy of publication. Some gene finders mentioned in the literature are now potentially lost to the public due to URLs changing or the program itself being taken offline.

Open source software has been a great gift to research. Nonetheless it comes with a price. Unlike commercial packages, there is no financial incentive to provide intensively tested programs with quality documentation. For the most part, and especially for specialised programs such as gene finders with a small user base, there is little or no user documentation, contact support is rare, and programs are error prone. There are certainly excellent exceptions to this bleak generalisation of open source software for gene finders and it has been an objective throughout the evaluation to find these exceptions.

The quality and quantity of data is indisputably one of the most important factors that impact the accuracy of *ab initio* gene finders. No matter how accurate the gene finder might be, the computer adage ‘garbage in – garbage out’ holds true. The algorithms of the programs used in the evaluation require training data and hence these data-driven programs are only as accurate as the data used to train them [68]. Ideally, experimentally validated data should be used in the training data, although even experimental data has the potential to be incorrect e.g. flawed interpretation of the results or simply experimental errors.

As shown by the results, gene finders per se are hugely inaccurate and so finding novel proteins from a purely *an ab initio* approach is still a major challenge. It may be unrealistic to expect gene finders to precisely find real genes in a DNA sequence that is a mere abstract model of a complex biological system [10]. The precise number of genes is not known for even the most studied and characterised complex genome, the human one. The stumbling block appears to be the split nature of eukaryotic genes due to introns. It is possible that using only a series of four letters to model the DNA molecule excludes vital signalling information. The cellular machinery can apparently recognise and process signals within the primary DNA sequence and pre-mRNA with precision [41]. Despite almost 20 years of research there is still no computational approach that can match the cellular machinery and consistently predict the exact exon-intron structure of genes from the DNA sequence alone. Suggesting a completely alternative computational representation of DNA is not unreasonable in the light that precise gene finding may simply be impossible with the current ‘sequence of letters’ representation. It is hypothesised that the next major breakthrough may be gene decoding in a DNA model at the atomic level [69], [70].

A major limitation of using *ab initio* gene finders to discover novel proteins missed by laboratory techniques is instigated by the biological phenomenon of alternative splicing. Even if the exons within a novel gene region are precisely predicted, there is currently no precise computational method to determine which exons should be included in the transcript. Alternative splicing can turn thousands of genes into hundreds of thousands of different RNA messages. Post-translational modifications can in turn create millions of different proteins. Millions of proteins can interact in complex biological networks to form hundreds of millions of metabolic pathways that ultimately affect the phenotype of the organism. Therefore to put things into perspective, even if the challenge of precisely finding exons in genes is realised, determining the final mature protein is an equally major challenge. In the evaluation presented in the paper *all* exons were included in the translation process. That is, there was no alternative splicing – only one protein sequence was obtained per gene prediction and it contained all exons. For future work, an insight into prevalence and patterns of alternative splicing in *T.gondii* genes may be achieved by generating predicted protein sequences for all possible exon inclusion/exclusion configurations and then performing homology searches.

The evaluation involved finding novel genes within the DNA sequence of *one* genome of a single strain of *T.gondii.* A genome sequence of a single strain does not indicate the genetic variability of a species [71]. The obvious approach to address genetic variability of a species is to use multiple genome sequences (from multiple strains of a single species). However, in their paper Mora and colleagues [71] from research in bacteria state that “mathematical extrapolation of existing data predicts that no matter how many strains have been sequenced, each sequence would contain genes that have not been encountered before”. The potential genetic variability may not be as extreme in eukaryotic pathogens but this extrapolation is an indication that there is still a long way to go before the pan-genome and the pan-proteome of all eukaryotic pathogen species is truly captured.

### Conclusion

This paper presented an evaluation of high-throughput *ab initio* gene finders with the intention of answering the question of whether existing bioinformatics tools can accurately discover proteins encoded in eukaryotic pathogen genomes. Whilst not too undermine the enormous effort in the *ab initio* gene finders developed so far, we conclude that the predicted exons are of insufficient accuracy to be used with confidence in the discovery of proteins missed by laboratory techniques. That is, their predicted exon locations are unreliable in the absence of experimental evidence. Precise exon locations are required for the successful translation to amino acid sequences. Also, the need for precise exon boundary delineation is equally important for isolating exons for alternative transcripts.

The gene finders perform reasonably well in locating the genomic location of the gene and it is possible to use a pool of gene finders to identify the approximate location of *every* gene encoded in the genome. In other words, candidate novel genes can be identified using gene finder consensus. Then, a possible exon-intron structure for these candidate novel genes can be manually determined from a consensus of their predicted exons. We hypothesise that knowing at least a crude sequence of a potential novel protein may help direct the experimental design to discover the real protein, and its location and function.

The accuracy of gene finding will progressively increase as the improvements in the quality of sequence data and computational techniques will inevitably occur. But it is debatable whether gene finding will ever be an exact science using the current DNA sequence model. Consequently, in order to exploit the expected explosion in the number of sequenced genomes, the challenge remains to develop *ab initio* gene finders that can find all genes, precisely identify their exon-intron structures, and handle large multiple genomes in a timely manner.

## Supporting Information

Supporting Information S1 Contains background information on gene prediction and gene finder programs.
(PDF)

## References

[1] Serruto D, Serino L, Masignani V, Pizza M (2009) Genome-based approaches to develop vaccines against bacterial pathogens. Vaccine 27: 3245–3250. 19200820 10.1016/j.vaccine.2009.01.072

[2] Kassahn KS, Waddell N, Grimmond SM (2011) Sequencing transcriptomes in toto. Integrative Biology 3: 522–528. 21298135 10.1039/c0ib00062k

[3] Wang Z, Gerstein M, Snyder M (2009) RNA-Seq: a revolutionary tool for transcriptomics. Nature Reviews Genetics 10: 57–63. 10.1038/nrg2484 PMC2949280 19015660

[4] Claverie J-M, Poirot O, Lopez F (1997) The difficulty of identifying genes in anonymous vertebrate sequences. Computers & Chemistry 21: 203–214. 9415985 10.1016/s0097-8485(96)00039-3

[5] Fickett JW (1996) The gene identification problem: An overview for developers. Computers and Chemistry 20: 103–118. 16749184 10.1016/s0097-8485(96)80012-x

[6] Fickett JW (1996) Finding genes by computer: the state of the art. Trends in Genetics 12: 316–320. 8783942 10.1016/0168-9525(96)10038-x

[7] Guigó R (1997) Computational gene identification: an open problem. Computers & Chemistry 21: 215–222. 9415986 10.1016/s0097-8485(97)00008-9

[8] Guigó R, Agarwal P, Abril JF, Burset M, Fickett JW (2000) An Assessment of Gene Prediction Accuracy in Large DNA Sequences. Genome Research 10: 1631–1642. 11042160 10.1101/gr.122800 PMC310940

[9] Korf I (2004) Gene finding in novel genomes. BMC Bioinformatics 5: 59. 15144565 10.1186/1471-2105-5-59 PMC421630

[10] Mathé C, Sagot MF, Schiex T, Rouzé P (2002) Current methods of gene prediction, their strengths and weaknesses. Nucleic Acids Research 30: 4103–4117. 12364589 10.1093/nar/gkf543 PMC140543

[11] Bork P (2000) Powers and Pitfalls in Sequence Analysis: The 70% Hurdle. Genome Research 10: 398–400. 10779480 10.1101/gr.10.4.398

[12] Ivanov IP, Firth AE, Michel AM, Atkins JF, Baranov PV (2011) Identification of evolutionarily conserved non-AUG-initiated N-terminal extensions in human coding sequences. Nucleic Acids Research 39: 4220–4234. 21266472 10.1093/nar/gkr007 PMC3105428

[13] Takahashi K, Maruyama M, Tokuzawa Y, Murakami M, Oda Y, et al (2005) Evolutionarily conserved non-AUG translation initiation in NAT1/p97/DAP5 (EIF4G2). Genomics 85: 360–371. 15718103 10.1016/j.ygeno.2004.11.012

[14] Touriol C, Bornes S, Bonnal S, Audigier S, Prats H, et al (2003) Generation of protein isoform diversity by alternative initiation of translation at non-AUG codons. Biology of the Cell 95: 169–178. 12867081 10.1016/s0248-4900(03)00033-9

[15] Chambers I, Harrison PR (1987) A new puzzle in selenoprotein biosynthesis - selenocysteine seems to be encoded by the stop codon, UGA. Trends in Biochemical Sciences 12: 255–256.

[16] Brent MR (2007) How does eukaryotic gene prediction work? Nat Biotech 25: 883–885. 10.1038/nbt0807-883 17687368

[17] Burset M, Seledtsov IA, Solovyev VV (2000) Analysis of canonical and non-canonical splice sites in mammalian genomes. Nucleic Acids Research 28: 4364–4375. 11058137 10.1093/nar/28.21.4364 PMC113136

[18] Patel AA, Steitz JA (2003) Splicing double: Insights from the second spliceosome. Nature Reviews Molecular Cell Biology 4: 960–970. 14685174 10.1038/nrm1259

[19] Xu Y, Mural RJ, Einstein JR, Shah MB, Uberbacher EC (1996) GRAIL: A multi-agent neural network system for gene identification. Proceedings of the Ieee 84: 1544–1552.

[20] Parra G, Blanco E, Guigo R (2000) GeneID in Drosophila. Genome Research 10: 511–515. 10779490 10.1101/gr.10.4.511 PMC310871

[21] Snyder EE, Stormo GD (1993) Identification of coding regions in genomic DNA-sequences - an application of dynamic-programming and neural networks. Nucleic Acids Research 21: 607–613. 8441672 10.1093/nar/21.3.607 PMC309159

[22] Solovyev VV, Salamov AA, Lawrence CB (1995) Identification of human gene structure using linear discriminant functions and dynamic programming. Proceedings/International Conference on Intelligent Systems for Molecular Biology; ISMB International Conference on Intelligent Systems for Molecular Biology 3: 367–375. 7584460

[23] Kulp D, Haussler D, Reese MG, Eeckman FH (1996) A generalized hidden Markov model for the recognition of human genes in DNA. Proceedings/International Conference on Intelligent Systems for Molecular Biology; ISMB International Conference on Intelligent Systems for Molecular Biology 4: 134–142. 8877513

[24] Gelfand MS, Mironov AA, Pevzner PA (1996) Gene recognition via spliced sequence alignment. Proceedings of the National Academy of Sciences of the United States of America 93: 9061–9066. 8799154 10.1073/pnas.93.17.9061 PMC38595

[25] Salamov AA, Solovyev VV (2000) Ab initio gene finding in Drosophila genomic DNA. Genome Research 10: 516–522. 10779491 10.1101/gr.10.4.516 PMC310882

[26] Burge C, Karlin S (1997) Prediction of complete gene structures in human genomic DNA. Journal of Molecular Biology 268: 78–94. 9149143 10.1006/jmbi.1997.0951

[27] Krogh A (1997) Two methods for improving performance of an HMM and their application for gene finding; Gaasterland TKPKKOCSCVA, editor. 179–186 p. 9322033

[28] Birney E, Durbin R (1997) Dynamite: a flexible code generating language for dynamic programming methods used in sequence comparison. Proceedings/International Conference on Intelligent Systems for Molecular Biology; ISMB International Conference on Intelligent Systems for Molecular Biology 5: 56–64. 9322016

[29] Lukashin AV, Borodovsky M (1998) GeneMark.hmm: New solutions for gene finding. Nucleic Acids Research 26: 1107–1115. 9461475 10.1093/nar/26.4.1107 PMC147337

[30] Yeh RF, Lim LP, Burge CB (2001) Computational inference of homologous gene structures in the human genome. Genome Research 11: 803–816. 11337476 10.1101/gr.175701 PMC311055

[31] van Baren MJ, Koebbe BC, Brent MR (2002) Using N-SCAN or TWINSCAN to Predict Gene Structures in Genomic DNA Sequences. Current Protocols in Bioinformatics: John Wiley & Sons, Inc. 10.1002/0471250953.bi0408s20 18428682

[32] Howe KL, Chothia T, Durbin R (2002) GAZE: A Generic Framework for the Integration of Gene-Prediction Data by Dynamic Programming. Genome Research 12: 1418–1427. 12213779 10.1101/gr.149502 PMC186661

[33] Hubbard T, Barker D, Birney E, Cameron G, Chen Y, et al (2002) The Ensembl genome database project. Nucleic Acids Research 30: 38–41. 11752248 10.1093/nar/30.1.38 PMC99161

[34] Majoros WH, Pertea M, Salzberg SL (2004) TigrScan and GlimmerHMM: two open source ab initio eukaryotic gene-finders. Bioinformatics 20: 2878–2879. 15145805 10.1093/bioinformatics/bth315

[35] Stanke M, Steinkamp R, Waack S, Morgenstern B (2004) AUGUSTUS: a web server for gene finding in eukaryotes. Nucleic Acids Research 32: W309–W312. 15215400 10.1093/nar/gkh379 PMC441517

[36] Gross S, Brent M (2006) Using Multiple Alignments to Improve Gene Prediction. Journal of Computational Biology 13: 379–393. 16597247 10.1089/cmb.2006.13.379

[37] Wei CC, Brent MR (2006) Using ESTs to improve the accuracy of de novo gene prediction. BMC Bioinformatics 7. 10.1186/1471-2105-7-327 PMC1534067 16817966

[38] DeCaprio D, Vinson JP, Pearson MD, Montgomery P, Doherty M, et al (2007) Conrad: Gene prediction using conditional random fields. Genome Research 17: 1389–1398. 17690204 10.1101/gr.6558107 PMC1950907

[39] Gross SS, Do CB, Sirota M, Batzoglou S (2007) CONTRAST: a discriminative, phylogeny-free approach to multiple informant de novo gene prediction. Genome Biology 8. 10.1186/gb-2007-8-12-r269 PMC2246271 18096039

[40] Schweikert G, Behr J, Zien A, Zeller G, Ong CS, et al (2009) mGene.web: a web service for accurate computational gene finding. Nucleic Acids Research 37: W312–W316. 19494180 10.1093/nar/gkp479 PMC2703990

[41] Baxevanis AD (2002) Predictive Methods Using DNA Sequences. Bioinformatics: John Wiley & Sons, Inc. 233–252.

[42] Borodovsky M, Rudd KE, Koonin EV (1994) Intrinsic and extrinsic approaches for detecting genes in a bacterial genome. Nucleic Acids Research 22: 4756–4767. 7984428 10.1093/nar/22.22.4756 PMC308528

[43] Parra Gs, Agarwal P, Abril JF, Wiehe T, Fickett JW, et al (2003) Comparative Gene Prediction in Human and Mouse. Genome Research 13: 108–117. 12529313 10.1101/gr.871403 PMC430976

[44] Margulies EH, Birney E (2008) Approaches to comparative sequence analysis: towards a functional view of vertebrate genomes. Nature Reviews Genetics 9: 303–313. 10.1038/nrg2185 18347593

[45] Montoya JG, Liesenfeld O (2004) Toxoplasmosis. The Lancet 363: 1965–1976. 10.1016/S0140-6736(04)16412-X 15194258

[46] Che F-Y, Madrid-Aliste C, Burd B, Zhang H, Nieves E, et al.. (2010) Comprehensive proteomic analysis of membrane proteins in toxoplasma gondii. Molecular & Cellular Proteomics. 10.1074/mcp.M110.000745 PMC3013445 20935347

[47] Kim K, Weiss LM (2004) Toxoplasma gondii: the model apicomplexan. International Journal for Parasitology 34: 423–432. 15003501 10.1016/j.ijpara.2003.12.009 PMC3086386

[48] Roos DS, Crawford MJ, Donald RG, Fohl LM, Hager KM, et al (1999) Transport and trafficking: Toxoplasma as a model for Plasmodium. Novartis Foundation symposium 226: 176. 10645546 10.1002/9780470515730.ch13

[49] Roos DS (2005) Themes and variations in apicomplexan parasite biology. Science 309: 72–73. 15994520 10.1126/science.1115252

[50] Stanke M, Tzvetkova A, Morgenstern B (2006) AUGUSTUS at EGASP: using EST, protein and genomic alignments for improved gene prediction in the human genome. Genome Biology 7: S11. 16925833 10.1186/gb-2006-7-s1-s11 PMC1810548

[51] Pertea M, Salzberg SL (2002) Using GlimmerM to Find Genes in Eukaryotic Genomes: John Wiley & Sons, Inc. 10.1002/0471250953.bi0404s00 18792941

[52] Sleator RD (2010) An overview of the current status of eukaryote gene prediction strategies. Gene 461: 1–4. 20430068 10.1016/j.gene.2010.04.008

[53] Kissinger JC, Gajria B, Li L, Paulsen IT, Roos DS (2003) ToxoDB: accessing the Toxoplasma gondii genome. Nucleic Acids Research 31: 234–236. 12519989 10.1093/nar/gkg072 PMC165519

[54] Burset M, Guigo R (1996) Evaluation of gene structure prediction programs. Genomics 34: 353–367. 8786136 10.1006/geno.1996.0298

[55] Guigo R, Flicek P, Abril JF, Reymond A, Lagarde J, et al.. (2006) EGASP: the human ENCODE genome annotation assessment project. Genome Biology 7. 10.1186/gb-2006-7-s1-s2 PMC1810551 16925836

[56] Dybas JM, Madrid-Aliste CJ, Che F-Y, Nieves E, Rykunov D, et al (2008) Computational Analysis and Experimental Validation of Gene Predictions in Toxoplasma gondii. PLoS ONE 3: e3899. 19065262 10.1371/journal.pone.0003899 PMC2587701

[57] Liu Q, Crammer K, Pereira FCN, Roos DS (2008) Reranking candidate gene models with cross-species comparison for improved gene prediction. BMC Bioinformatics 9. 10.1186/1471-2105-9-433 PMC2587481 18854050

[58] Knapp K, Chen Y-PP (2007) An evaluation of contemporary hidden Markov model genefinders with a predicted exon taxonomy. Nucleic Acids Research 35: 317–324. 17170005 10.1093/nar/gkl1026 PMC1802560

[59] Allen JE, Pertea M, Salzberg SL (2004) Computational Gene Prediction Using Multiple Sources of Evidence. Genome Research 14: 142–148. 14707176 10.1101/gr.1562804 PMC314291

[60] Haas B, Salzberg S, Zhu W, Pertea M, Allen J, et al (2008) Automated eukaryotic gene structure annotation using EVidenceModeler and the Program to Assemble Spliced Alignments. Genome Biology 9: R7. 18190707 10.1186/gb-2008-9-1-r7 PMC2395244

[61] Allen JE, Salzberg SL (2005) JIGSAW: integration of multiple sources of evidence for gene prediction. Bioinformatics 21: 3596–3603. 16076884 10.1093/bioinformatics/bti609

[62] Liu Q, Mackey AJ, Roos DS, Pereira FCN (2008) Evigan: a hidden variable model for integrating gene evidence for eukaryotic gene prediction. Bioinformatics 24: 597–605. 18187439 10.1093/bioinformatics/btn004

[63] Lomsadze A, Ter-Hovhannisyan V, Chernoff YO, Borodovsky M (2005) Gene identification in novel eukaryotic genomes by self-training algorithm. Nucleic Acids Research 33: 6494–6506. 16314312 10.1093/nar/gki937 PMC1298918

[64] Li L, Brunk BP, Kissinger JC, Pape D, Tang KL, et al (2003) Gene discovery in the Apicomplexa as revealed by EST sequencing and assembly of a comparative gene database. Genome Research 13: 443–454. 12618375 10.1101/gr.693203 PMC430278

[65] Wakaguri H, Suzuki Y, Sasaki M, Sugano S, Watanabe J (2009) Inconsistencies of genome annotations in apicomplexan parasites revealed by 5′-end-one-pass and full-length sequences of oligo-capped cDNAs. BMC Genomics 10. 10.1186/1471-2164-10-312 PMC2722674 19602295

[66] Wastling JM, Xia D, Sohal A, Chaussepied M, Pain A, et al (2009) Proteomes and transcriptomes of the Apicomplexa - Where’s the message? International Journal for Parasitology 39: 135–143. 18996390 10.1016/j.ijpara.2008.10.003

[67] Harrow J, Nagy A, Reymond A, Alioto T, Patthy L, et al.. (2009) Identifying protein-coding genes in genomic sequences. Genome Biology 10. 10.1186/gb-2009-10-1-201 PMC2687780 19226436

[68] Flower DR, Macdonald IK, Ramakrishnan K, Davies MN, Doytchinova IA (2010) Computer aided selection of candidate vaccine antigens. Immunome Research 6 Suppl 2S1. 10.1186/1745-7580-6-S2-S1 PMC2981880 21067543

[69] Ninaber A, Goodfellow JM (1999) DNA conformation and dynamics. Radiation and Environmental Biophysics 38: 23–29. 10384952 10.1007/s004110050134

[70] Service RF (2000) DNA imaging - Getting a feel for genetic variations. Science 289: 27–28. 10928923 10.1126/science.289.5476.27a

[71] Mora M, Donati C, Medini D, Covacci A, Rappuoli R (2006) Microbial genomes and vaccine design: refinements to the classical reverse vaccinology approach. Curr Opin Microbiol 9. 10.1016/j.mib.2006.07.003 16890009

