# Supplementary material for: Evaluating High-Throughput Ab Initio Gene Finders to Discover Proteins Encoded in Eukaryotic Pathogen Genomes Missed by Laboratory Techniques
Source: PLoS One. 2012 Nov 30;7(11):e50609. doi: 10.1371/journal.pone.0050609 (PMC3511556; doi:10.1371/journal.pone.0050609)
Supplement: Supporting Information S1 — Contains background information on gene prediction and gene finder programs. (PDF) [file pone.0050609.s001.pdf]

Introduction

This is an additional document to the PLoS ONE article entitled, “Evaluating high-throughput *ab initio* gene finders to discover proteins encoded in eukaryotic pathogen genomes missed by laboratory techniques”. It contains three main sections: Gene Finder programs, Background Information, and Gene Finder commands. The document is intended to be a reference for gene prediction and is not expected to be read from cover-to-cover.

Gene Finder programs (page 1) contains a description of all gene finder programs used in the article. There is a set format for each program entry: Program Name, Usage, Description, Platform, Implementation, Algorithm, Availability, Licence, Version, First Released, References, Input, Output, Advantages, Disadvantages, and Comments. The programs appear in alphabetical order.

Background Information (page 7) contains basic introductory information that may assist the researcher in a better understanding of gene prediction.

Gene Finder commands (page 13) contains, for reference only, syntax commands for setting up and executing the gene finder programs.

Gene Finder Programs

AUGUSTUS

Program Name: augustus

Usage: Predicts genes in eukaryotic genomic sequences.

Description: The method is based on a **generalized Hidden Markov Model** with a new method for modelling the intron length distribution.

Platform: Web server or UNIX/Linux.

Implementation: C++ and Perl

Algorithms: Windowed weight array model (WWAM) and interpolated Markov Models (IMM)

Availability http://bioinf.uni-greifswald.de/augustus/

Licence: Open Source

Version: 2.6

First Released: 2004

Reference: Stanke M, Steinkamp R, Waack S, Morgenstern B: **AUGUSTUS: a web server for gene finding in eukaryotes**. *Nucleic Acids Research* 2004, **32**(suppl 2):W309-W312

Input: a DNA sequence in FASTA format or multiple sequences in multiple FASTA format and model parameters for target species

Output: A text output in the 'General Feature Format' (GFF)
(See http://www.sanger.ac.uk/resources/software/gff/)

To extract the protein sequences from the gff file a Perl script called getAnnoFasta.pl is provided.

Advantages: AUGUSTUS can be run on the German MediGRID. This enables you to submit larger sequence files and allows you to use protein homology information in the prediction.

Disadvantages:

Training:
AUGUSTUS has currently been trained on species specific training sets to predict genes in organisms (see http://augustus.gobics.de/) for the list of organisms. A training program called etraining is available and requires training genes and their exon locations to be in a single file in a genBank format. The main keys required are LOCUS heading (LOCUS entries are optional), FEATURES, source, gene, mRNA, CDS, and ORIGIN (followed by the DNA sequence containing the training genes). How to train AUGUSTUS can be found at:
http://molecularevolution.org/molevolfiles/exercises/augustus/training.html

Comments:
AUGUSTUS is based on a Hidden Markov Model and integrates a number of known methods and sub models e.g. Markov chain, a higher order windowed weight array model (WWAM), interpolated Markov Models (IMM) and a novel method for similarity-based weighting of sequence patterns. The default version of the model consists of 47 states (23 states for forward strand and 23 symmetric states for reverse strand). Each state emits a random DNA string of possibly random length. The distribution of these and the transition probabilities between them are determined using established models and a training set of annotated sequences for the target species. The following are probabilistically modelled separately: 1) the sequence around the splice sites, 2) the sequence of the branch point region, 3) the bases before the translation start, 4) the coding regions, 5) the non-coding regions, 6) the first coding bases of a gene, the length distribution of single exons, initial exons, 7) internal exons, 8) terminal exons, 9) intergenic regions,10) the distribution of the number of exons per gene, and 11) the length of distribution of introns. AUGUSTUS employs a new way of modelling intron lengths by combining explicit length modelling (estimated from observed frequencies) for short introns, with a geometric distribution for long introns. Short introns typically have a length distribution clustering around a certain length.

AUGUSTUS predicts the gene structure with the largest a-posteriori probability using the Viterbi-algorithm.

GeneMark.hmm eukaryotic version

Program Name: gmhmme3

Usage: A eukaryotic gene finding algorithm using hidden Markov models (HMM)

Description: GeneMark-hmm employ inhomogeneous (three-periodic) Markov chain models describing protein-coding DNA and homogeneous Markov chain models describing non-coding DNA. It utilises an extended hidden Markov model (HMM) architecture and the generalized Viterbi algorithm to determine the most likely sequence of hidden states (labels designating the coding or non-coding function) based on the whole observed DNA sequence. The hidden states are: initial, internal and terminal exons, introns, intergenic regions and single exon genes located on both DNA strands; and initiation site, termination site, donor and acceptor splice sites.

Platforms: Web server, Linux and Sun Solaris

Implementation:

Algorithms: Hidden Markov Model (HMM) with duration or a hidden semi-Markov model (HSMM).

Availability: http://exon.biology.gatech.edu/

Licence: Open Source Software

Version: 3.3

First Released: 1997

References:
Lukashin AV, Borodovsky M: **GeneMark.hmm: New solutions for gene finding**. *Nucleic Acids Research* 1998, **26**(4):1107-1115
Besemer J, Borodovsky M: **GeneMark: web software for gene finding in prokaryotes, eukaryotes and viruses**. *Nucleic Acids Research* 2005, **33**:W451-W454.

Input: a DNA sequence in FASTA format and model parameters for target species

Output: an option to output in the 'General Feature Format' (GFF) and an option to translate predicted DNA sequences to protein sequences.

Advantages:

Disadvantages: There is no training program. However, there is a self-training version of GeneMark (see below)

Trained model files: Model files for the following organisms are provided and have the extension *.mod:

Homo sapiens, Arabidopsis thaliana, Caenorhabditis elegans, Toxoplasma gondii, Chlamydomonas reinhardtii, Drosophila melanogaster, Gallus gallus, Hordeum vulgare, Mus musculus, Oryza sativa, Triticum aestivum and *Zea mays*

Comments: There are 2 versions of GeneMark.hmm - Supervised and un-supervised training versions. The version that uses supervised training has a web interface:
http://exon.biology.gatech.edu/eukhmm.cgi

GeneMark.hmm ES

Program Name: gm_es.pl

Usage: A eukaryotic gene finding algorithm using hidden Markov models (HMM) and employing the Viterbi unsupervised training procedure. ("E" stands for "Eukaryotic" and "S" stands for "Self-training")

Description: GeneMark.hmm-ES program predicts genes and intergenic regions in a sequence as a whole. They use the Hidden Markov models reflecting the "grammar" of gene organization. The self-training procedure determines parameters for the gene models

Platform: Linux and Sun Solaris

Implementation: Perl

Algorithm: Markov Model (GHMM) with Viterbi training

Availability: http://exon.biology.gatech.edu/

Licence: Open Source Software

Version: 1.0

First Released: 2005

Reference:
Lomsadze A., Ter-Hovhannisyan V., Chernoff Y. and Borodovsky M., "Gene identification in novel eukaryotic genomes by self-training algorithm", **Nucleic Acids Research**, 2005, Vol. 33, No. 20, 6494-6506

Input: a DNA sequence in FASTA format

Output: a file in a Gene Transfer Format (gtf).

Advantages: Can be used on novel genomes where there are an inadequate number of experimentally validated genes. The training set used is classified as "unsupervised training". GeneMark.hmm ES has been tested on *Toxoplasma gondii.*

Disadvantages: Contains no option to convert predicted genes to protein sequences

Comments:
GeneMark-ES tested on genomes of *Arabidopsis thaliana, Caenorhabditis elegans and Drosophila melanogaster.*

There is an optional parameter (--BP OFF) that switches off the branch point sub model and runs original ES algorithm (GeneMark.hmm ES version 1.0). This option is recommended for genomes with weak branch points and was used with *Toxoplasma gondii*

Generation of gene predictions for a novel eukaryotic genome occurs in parallel with the unsupervised (automatic) iterative estimation of gene model parameters by the Viterbi training. At each iteration, the algorithm takes genomic sequence labelled by the Viterbi algorithm at the previous iteration into coding and non-coding regions, re-estimates model parameters, and computes a new sequence parse and labelling. This general path of the iterative Viterbi training process is modified by addition of restrictions on possible changes of parameters to ensure convergence of the iteration process to the biologically relevant point. At the point of convergence the set of sequence labels is transformed into the list of gene predictions, the program output.

GlimmerHMM

Program Name: glimmerhmm_linux

Usage: A Generalized Hidden Markov Model **eukaryotic** gene-finder

Description: GlimmerHMM is a gene finder based on a Generalized Hidden Markov Model (GHMM). Although the gene finder conforms to the overall mathematical framework of a GHMM, additionally it incorporates splice site models adapted from the GeneSplicer program and a decision tree adapted from GlimmerM. It also utilizes Interpolated Markov Models for the coding and non-coding models.

Platform: Linux RedHat 6.x+, Sun Solaris, and Alpha OSF1

Implementation: C/C++

Algorithm: Generalized Hidden Markov Model (GHMM)

Availability: http://www.cbcb.umd.edu/software/glimmerhmm/

Licence: OSI Certified Open Source Software

Version: 3.0.1 (2007)

First Released: 2004

References:
Pertea M, Salzberg SL: **Using GlimmerM to Find Genes in Eukaryotic Genomes**: John Wiley & Sons, Inc.; 2002
Majoros WH, Pertea M, Salzberg SL: **TigrScan and GlimmerHMM: two open source ab initio eukaryotic gene-finders**. *Bioinformatics* 2004, **20**(16):2878-2879

Input: Two inputs - a DNA sequence file in FASTA format and a directory containing the training parameters for the program.

Output: A text output in the 'General Feature Format' (GFF)
(See http://www.sanger.ac.uk/resources/software/gff/)

Advantages: Code reusable due to their modular and extensible architectures. The programs are re-trainable by the end user. They are also re-configurable and include several types of probabilistic sub models which can be independently combined, such as Maximal Dependence Decomposition trees and interpolated Markov models.

Disadvantages: No option to convert the predicted DNA sequences to protein sequences.

Training: Training program is available

Comments:

GlimmerHMM was used for the annotation of the *Aspergillus fumigatus* and *Toxoplasma gondii* genomes.

Currently, the GHMM structure includes introns of each phase, intergenic regions, and four types of exons (initial, internal, final, and single).

SNAP

Program Name: snap

Usage: A Generalized Hidden Markov Model **eukaryotic** gene-finder. SNAP is an acronym for Semi-HMM-based Nucleic Acid Parser.

Description: SNAP models protein-coding sequences in genomic DNA via a specialized hidden Markov model similar to the one used in Genscan

Platform: A command line UNIX program

Implementation: ANSI C

Algorithm: Hidden Markov Model (HMM)

Availability: http://homepage.mac.com/iankorf/

Licence: GNU General Public License

Version: 2006-07-28

First Released: 2004

Reference: Korf I: **Gene finding in novel genomes**. *BMC Bioinformatics* 2004, **5**(1):59

Input: a DNA sequence in FASTA format and model parameters for target species

Output: an option to output in the 'General Feature Format' (GFF) and an option to translate predicted DNA sequences to protein sequences.

Advantage: Attempts to be more adaptable to different organisms, addressing problems related to using a gene finder on a genome sequence that it was not trained against.

Disadvantages:

Comments:
The default HMMs employed by SNAP have a minimal genome model. There are no models for promoter, poly-A site, UTRs or trans-splicing. These features are often not annotated and the author states that only the features which can be unambiguously defined in the training data should be used.

Some key SNAP features: 1) Uses six intron states to prevent stop codons at splice junctions, 2) models each strand independently. Decoding the strands independently allows genes on opposite strands to overlap. The advantage is that it allows genes within introns of other genes. The disadvantage is that it also allows overlapping exons. 3) The state diagram is read from a parameter file. This allows one to change the HMM to describe a variety of genomic features. 4) The sequence feature model architecture allows one to employ any length weight matrix and any order Markov model and to embed these models within an array, decision tree, or 3-periodic (coding sequence) framework. 6) Introns may have explicit length distributions over a fixed distance followed by a geometric tail (Similar to AUGUSTUS).

It may be possible to increase the accuracy of SNAP by including more states in the HMM to model additional genomic features or by using more sophisticated statistical techniques such as interpolated Markov models, maximum dependence decomposition trees or isochore segmentation.

SNAP employs weight matrices (WM) to model compositional features such as the translation start site, splice sites and codon bias. The "default" models are as follows: the acceptor WM is30 bp long with 3 exonic nucleotides, the donor WM is 9 bp with 3 exonic nucleotides, the start WM is 12 bp with 6 coding nucleotides, and the stop WM is 9 bp with 3 bp on either side of the stop codons. Markov models are used for exons, introns, and intergenic sequence.

Background Information

The following is a brief introduction to *in silico* gene finding. In particular, the focus is on *ab initio* gene finding that predicts genes within a DNA sequence with no additional evidence. Figure 1 shows the molecular steps from a DNA sequence containing a single gene to a polypeptide sequence: Step 1) DNA sequence is transcribed to produce a messenger RNA (mRNA). In effect the introns (the non-coding sequences) are removed due to splicing and the exons (the coding sequences) are concatenated; and step 2) mRNA is translated into a chain of amino acids (a polypeptide) that folds to form a protein. These steps are commonly referred to as the central dogma of molecular biology.

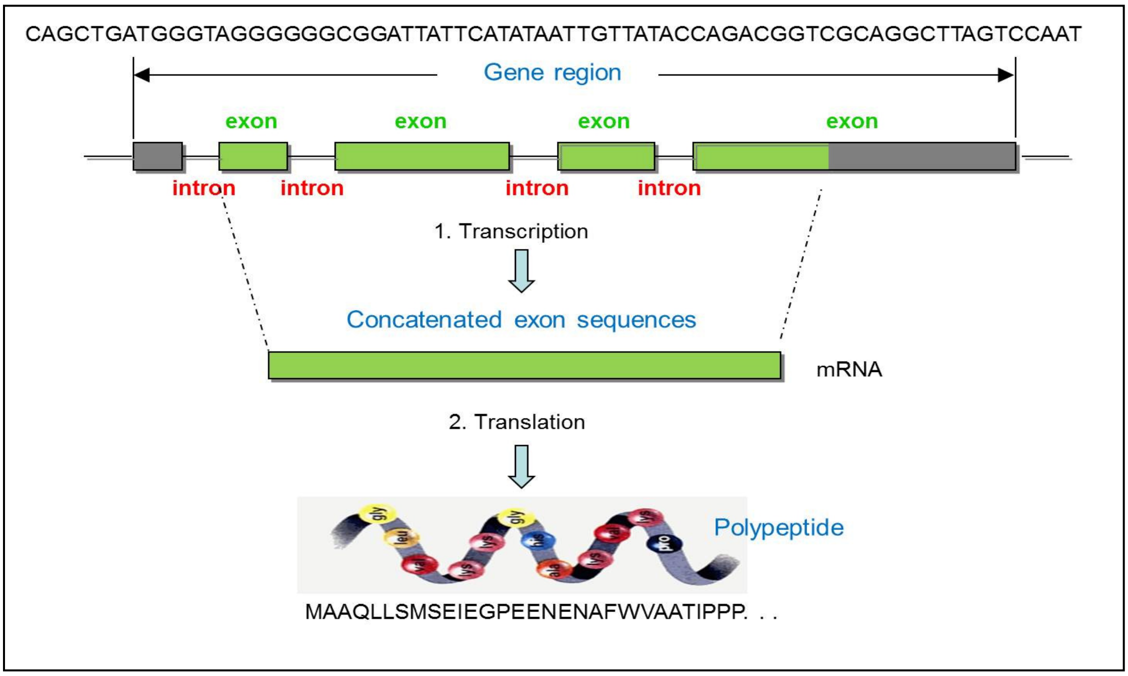

Figure 1. Central dogma of molecular biology

The general aim in gene finding is to identify the coding segment (CDS) within a gene region; but more specifically, identify exons within the coding segment. Once the internal exon-intron structure of a gene is known, the encoded protein(s) can be deduced. At the most basic understanding there are three types of exons: an initial exon that is defined by a start codon (typically ATG), an internal exon, and a terminal exon that is defined by a stop codon (typically TAG, TGA, or TAA).

Figure 2 shows a schematic of the gene finder motif signals. In addition to the start and stop codon signals in the DNA sequence of the CDS, an intron begins with a splice donor site (typically GT) and ends with a splice acceptor site (typically AG). Hence, there are four basic motif signals defining coding exons: start and stop codon, and donor and acceptor sites. These motif signals define the boundaries for the exon-intron structure of a gene and in effect define the syntax rules for parsing the DNA sequence. For example, valid gene parses are ATG → TGA, ATG →GT → AG → TGA. An invalid gene parse is ATG → AG. Some valid parses will not be true genes because not all ATGs code for a start codon, and not all GTs and AGs are splice sites,

Given a single DNA sequence as input, gene finders attempt to predict for each base along the sequence whether it is part of an exon, intron, or intergenic region. The basic steps for predicting genes are: 1) identify all possible boundary motif signals, 2) link the boundary signals based on syntax rules into valid gene parses, 3) score all valid gene parses, and 4) output the gene predictions with the highest scoring parses.

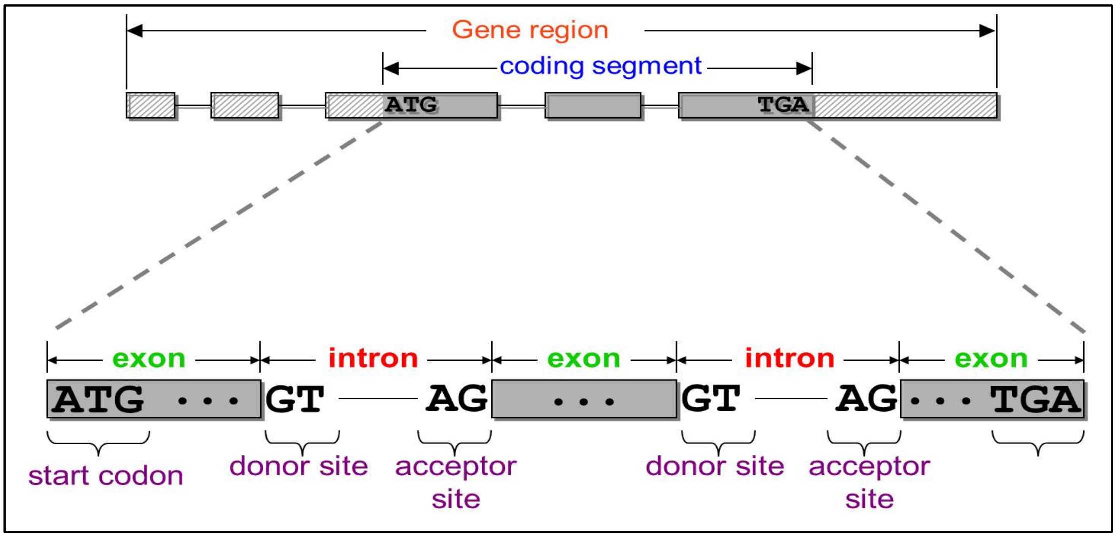

Figure 2. Gene finder motif signals (Source: Majoros WH, *Methods for Computational Gene Prediction*, Cambridge University Press)

Methods from the fields of natural language processing and speech recognition have been adopted in computational gene finding. Instead of identifying features such as a subject or a verb in a sentence, features of biological significance in a DNA sequence are identified. There are two broad classes of features representing DNA subsequences: 1) Motif Signals - short, fixed-length features such as start and stop codons, and splice sites; and 2) Content regions - variable-length features such as exons and introns. Codon bias is one natural phenomenon that can help predict the content regions. That is, some codons in protein coding regions are significantly more common than other codons in non-coding regions. *Ab initio* gene prediction searches for signals and content. Figure 3 shows the signal and content sensors. In signal sensing, a sliding window identifies and scores the motif signals along the DNA sequence. Typically, position-specific weight matrices (PWMs) are employed in the sequence signal detection. The parameters of the PWM are estimated from known signals of the same type as part of the training process. The sliding window which constitutes a PWM for a specific signal (e.g. start codon) can have any fixed number of cells. For example, in Figure 3 this sliding window has nine cells, four cells prior to and two cells after the signal consensus (ATG). Each cell of the PWM contains the frequency distribution of each of the four nucleotides. For example, the fifth, sixth and seventh cells in Figure 3 have the following nucleotide distribution: (A = 1.0, C = 0.0, G = 0.0, T= 0.0) (A = 0.0, C = 0.0, G = 0.0, T= 1.0) (A = 0.0, C = 0.0, G = 1.0, T= 0.0). All other six cells have a distribution relative to the compositional biases at the corresponding positions flanking start codons (ATGs) in known genes of the target organism. That is, computational biases are *learned* from the process of parameter estimation, or training. Validated genes from the organism of interest comprise the training data, or training set. Each organism has its own compositional biases. The PWM window

slides along the DNA sequence one nucleotide at a time. A signal score is computed at each location by multiplying the cell probabilities of the observed nucleotides in each cell of the window. The probabilities are derived from the estimated parameters of the PWM. Taking Figure 3 as an example, the score at the shown location for this nine celled sliding window is the probability of G in cell one multiplied by the probability of T in cell two, and so on. Only signals with scores over a predetermined threshold are selected for the next phase of the gene finding process, which involves content sensing.

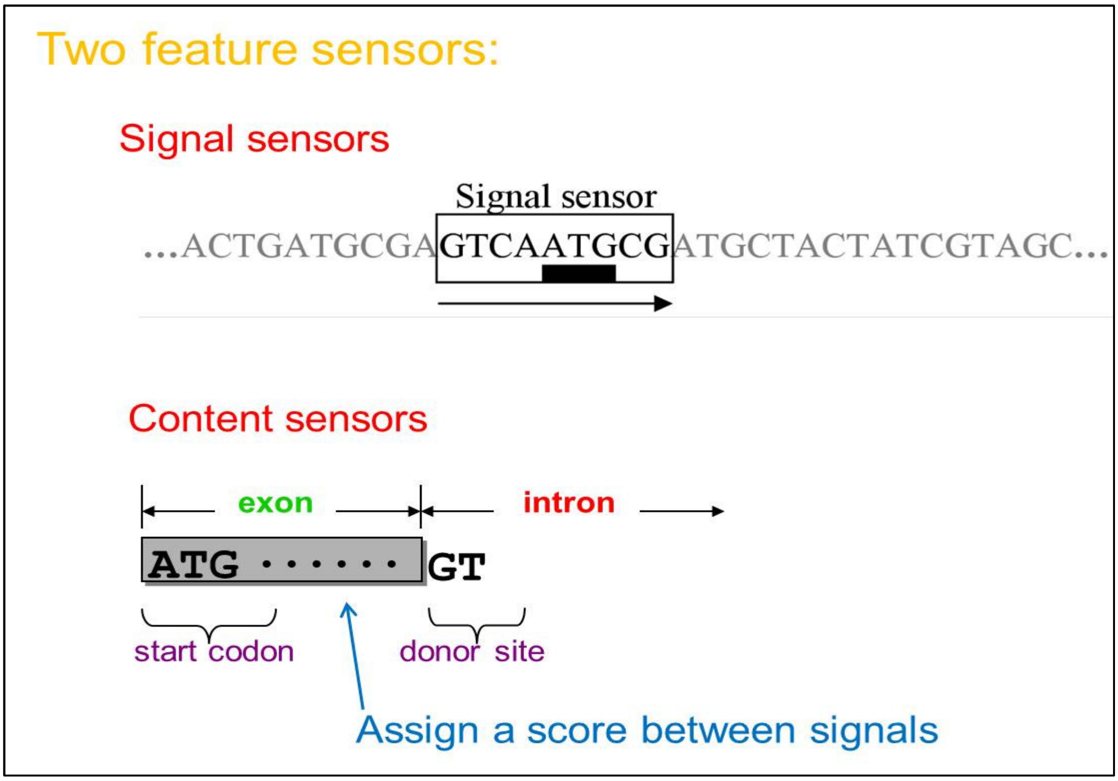

Figure 3. Feature sensors for ab initio gene finding

Content sensing is the process of scoring the regions between the motif signals in a valid parse, such as exons, introns, or intergenic regions. The regions are scored in accordance with content measures (also referred to as codon statistics). The current most popular content-based measure is in the form of a Markov chain (or model). For example, it is a model that predicts the next base in a sequence using a fixed number of preceding bases. A more specific example is hexamer frequencies in the form of codon position-dependent fifth-order Markov models. It uses the five previous bases to predict the next base. The signal and content scores are combined to determine which putative exons are ultimately included in the overall predicted gene structure.

A Generalized hidden Markov model (GHMM) provides a framework to integrate signal scores and content scores. Figure 4 shows a state-transition diagram of a GHMM. Each gene feature corresponds to a state in diagram. The signal features are represented by circle states and the content regions by the oval states. Each state has its own emission probability distribution and emits a subsequence of the input sequence. For example, in the figure the donor state emission distribution is shown and GT is the most probable sequence emitted from this state. An input sequence can be scored by assessing the emission probabilities for a particular valid parse (or path through the GHMM model). The emission probabilities collectively comprise signal scores (produced from the circle states) and

content scores (produced from the oval states). To score the variable length of the content regions (i.e. the exons, introns, and intergenic regions), each content state generates an additional distribution of duration probabilities. In other words, GHMM (but not HMM) explicitly model feature lengths of exons and to a lesser extent introns and this is important where feature lengths do not follow a geometric distribution.

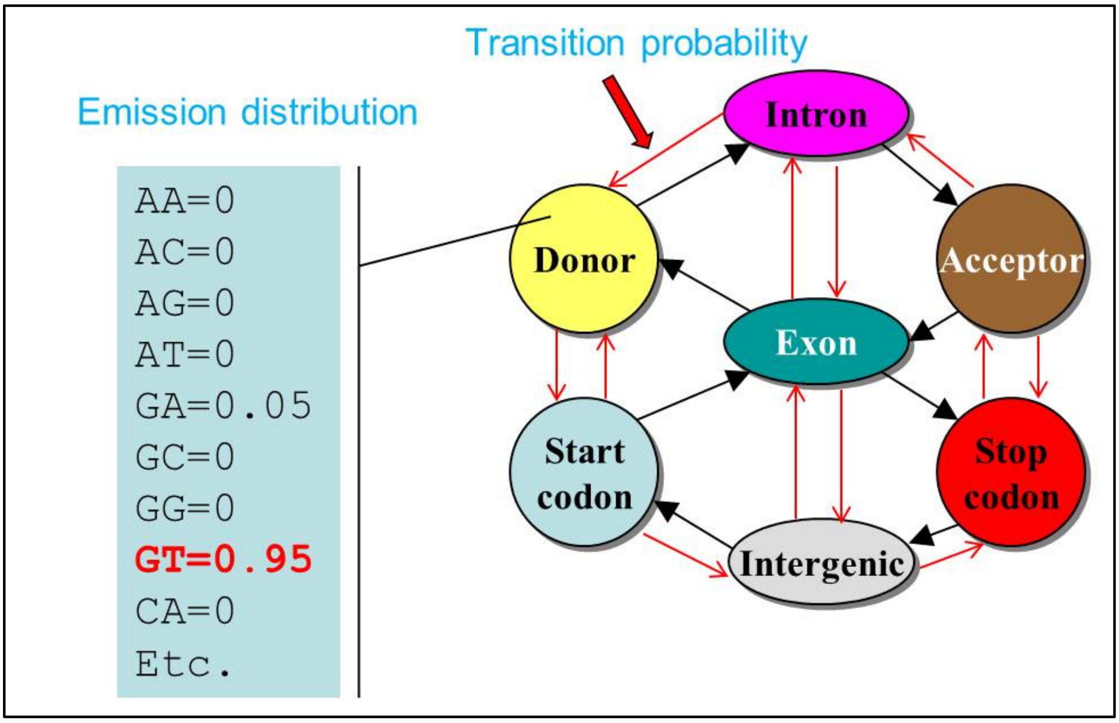

Figure 4. A state-transition diagram of a generalized hidden Markov model (GHMM)

Each state has fixed transition probabilities from one state to another state (represented in diagram by arrows). The transition probabilities describe the linear order in which the states are expected to occur. In this example, the red arrows are the low transition probabilities and the black arrows the high transition probabilities. In effect the transitions define the syntax rules for parsing the DNA sequence.A general rule is that the states representing the content regions (the oval states) cannot transition directly to each other. Each state has its own separate sub-model or sensor (see Figure 3). The signal sensor uses the emission distribution to detect the appropriate motif signal and the content sensor assigns a probability score between two signals to determine the subsequence to be emitted by the state.

Figure 5 shows how a GHMM model is fitted to an input DNA sequence to predict exons. For example, let us assume we are at the intergenic state. Each state emits a subsequence based on its emission probability distribution. The probabilities are derived from the training sequences for which the correct gene structure is known. We choose which state to visit next based on the transition probability (in the figure the low transition probabilities have been removed for clarity). The path through the states is referred to as a Markov chain – meaning that the state we go to next depends on the state that we are in. Since only the observed input sequence is given, the actual state path is *hidden*

and needs to be inferred. Each nucleotide in the input sequence can only be emitted from only one state. The model determines the probability that a nucleotide was emitted by a particular state.

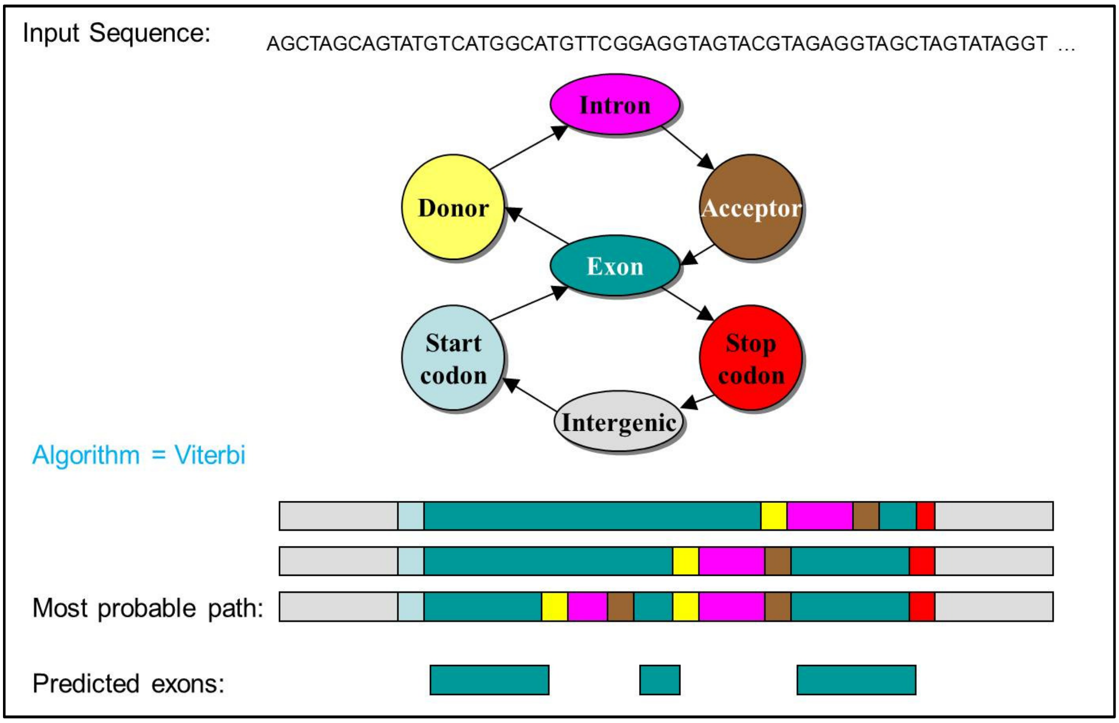

Figure 5. Predicting exon location with generalized hidden Markov model (GHMM)

Only three putative paths (represented by a series of coloured rectangles) are shown below the state-transition diagram. The colours of the rectangles correspond to the colours of the states e.g. exons are green.

There are potentially many state paths (putative gene parses) that could emit the same sequence. To evaluate and score *all* possible valid parses is too time consuming. In practice, a shortest path algorithm (incorporating dynamic-programming) is used to find the highest-scoring parse for the input sequence (i.e. the path with the highest probability). The term *decoding* is used to describe the problem of identifying the highest-scoring parse. Decoding is typically achieved by an algorithm called Viterbi. Figure 6 shows a summary of the steps involved in predicting protein sequences.

Prediction accuracy depends on both the number of genes and the variety of genes in the training set. A training set in effect represents an “average gene” so it can be expected that some genes in the genome will not be predicted or will be incorrectly predicted because they greatly differ in structure or compositional biases to the so-called average gene. In mammalian genomes, there can be regions in the genome that are rich or poor in GC content in comparison to regions in the rest of the genome. These regions are called isochores and tend to have more functional genes. It is proposed that gene prediction accuracy in mammalian genomes will increase if the compositional biases are modelled for each isochore.

Most gene finders for efficiency make the following assumptions, which may be invalid at times:

1) The motif signals (start and stop codons, and splice sites) are the same throughout the genome;
2) The lengths for the introns and intergenic regions are geometrically distributed (AUGUSTUS is an exception);

3) Genes do not overlap;
4) Genes are not nested within other genes;
5) The input sequence contains no sequencing errors;
6) There is no alternative splicing .i.e. only one single, highest scoring isoform per gene is predicted; and
7) The start and stop codons contain no introns.

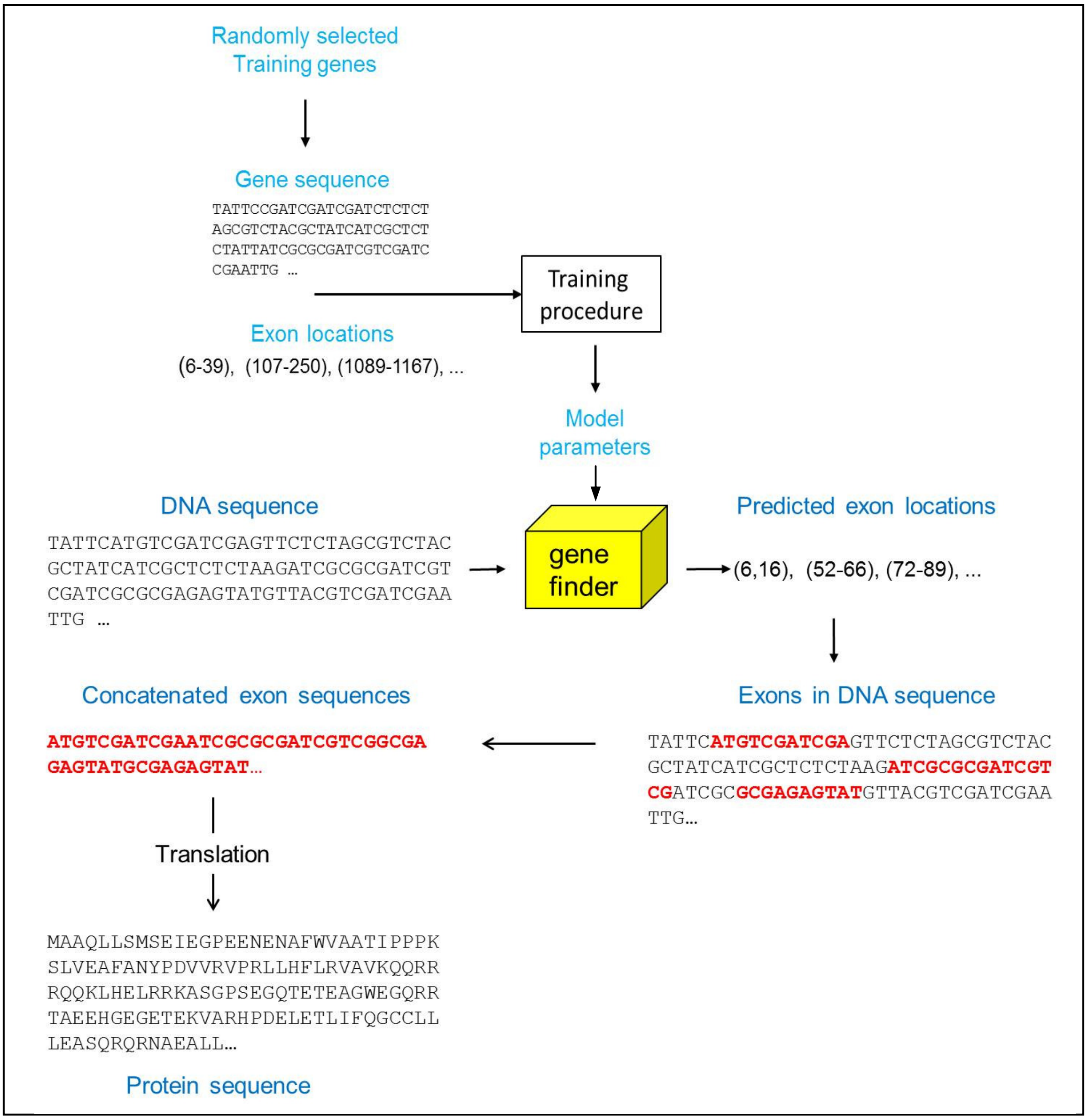

Figure 6. Predicting protein sequences

This schematic shows a summary of the steps involved in predicting protein sequences when given a DNA sequence as input. The first major step is the creation of model parameters (the determination of the probability distributions) for the respective gene finder. This is achieved by using validated genes from the target pathogen as training genes. The more genes used the greater the prediction accuracy but also the greater the potential for overtraining. The training procedure requires two input files: one containing the validated gene sequences and the other the exon locations of these gene sequences.

The model parameters along with the DNA sequence, in which we want to predict genes, are the only input requirements to the gene finder. One of the primary outputs is a file containing all the predicted exon locations per predicted gene. Using the exon start and end coordinates (e.g. start = 6 and end = 16) the exon nucleotide sequences (shown in red) are extracted from the input DNA sequence. These exon sequences are concatenated to form one sequence per gene prediction and in effect are equivalent to an mRNA sequence without the 5´cap or polyA tail. Commencing at the start of the mRNA sequence three letter codons (e.g. ATG,TCG, ATC) are translated to amino acid letters (e.g. M,S, I) to construct the protein sequence

References

Baxevanis, A. D., and Ouellete, B. F. *Bioinformatics, Third Edition*. John Wiley and Sons, 2005

Edwards, D., Stajich J., and Hansen D. *Bioinformatics – Tools and Applications*. Springer, 2009

Majoros, W. H. *Methods for Computational Gene Prediction*. Cambridge University Press, 2007

Gene Finder Commands

For the gene prediction programs we need to know the exon start and end locations for the training data genes. The main place to find this information is in GenBank under the CDS join section as shown below:

*CDS join(22502..26539,27124..27259,28110..30498,31038..31104,
31285..31575,31925..32903,33391..35489,36324..36458,
36903..37039,37723..37822,38124..38225,38690..38821,
39431..39811,40299..41876)*

Each exon is represented by two numbers separated by "..". The first number e.g. 25502 is the start coordinates of the exon and the second number e.g. 26539 is the end coordinates of the exon. The exons are list in the order that they occur in the gene.

```
####### Syntax commands for setting up and executing the gene finders ############
#
#	The following are the commands to run the various Gene finder programs.
#	Lines that commence with a # are comments and any blank line are ignored.
#	Lines without a # are commands.
#
#	The commands are in the relevant order which means the entire file can in theory be
	executed as a Linux script to consecutively run each command.
#	e.g.	./script_example
#
#	Alternatively the commands can be copied and ran one at a time
#
#	This file has been configured to process an input file in a FASTA format containing
#	 the nucleotide sequences of the target pathogen.
#
#	In this example the target pathogen = Toxoplasma gondii and the input file is called
#	TGME49_chrVIIb.txt contains the sequence for the chromosome VIIb.
#	In theory the user only needs to change the input and output filenames, and the path
#	location of the executables and input files. However, the intention here is more to aid the user
#	by providing relevant commands that can be copied and edited for the user's specific setup
#	environment.
######################################################################

## BLAST installation ###

# Download BLAST + version for LINUX
lwp-download ftp://ftp.ncbi.nlm.nih.gov/blast/db/ncbi-blast-2.2.25+-ia32-linux.tar.gz

# Decompress tar file
tar -xvpf ncbi-blast-2.2.25+-ia32-linux.tar.gz
```

```
# Append to the path so that BLAST programs will run from any directory
PATH=$PATH:$HOME/Blast/ncbi-blast-2.2.25+/bin
export PATH
echo PATH

# Alternatively can add the following two lines to .bash_profile
PATH=$PATH:$HOME/Blast/ncbi-blast-2.2.25+/bin
export PATH

# Download appropriate databases for BLAST
lwp-download ftp://ftp.ncbi.nlm.nih.gov/blast/db/nr.00.tar.gz nr.00.tar.gz

# Alternatively use Perl script update_blastdb.pl to download pre-formatted BLAST databases from
# NCBI

perl update_blastdb.pl --showall      #Show all available pre-formatted BLAST databases

# Download non-redundant protein sequence database with entries from GenPept, Swissprot, PIR,
# PDF, PDB, and NCBI RefSeq
perl update_blastdb.pl -d nr

# Large databases are formatted in multiple 1 Gigabytes volumes e.g. non-redundant protein sequence
# database, which are named using the database.##.tar.gz convention. All relevant volumes are
# required. An alias file is provided so that the database can be called using the alias name without the
# extension when using BLAST. For example, to call nr database, simply use "-d nr" option in the
# commandline

# Decompress the multiple database tar files
for file in `ls nr.*.tar.gz`;do tar -xzf $file;done

# Create a file called .ncbirc (a blast configuration file) in /home directory and add the following 2
# lines. These 2 lines direct the blast programs were to find the databases:
[BLAST]
BLASTDB=$HOME/Blast/db

# Use the following to extract a sequence from a BLAST database in a FASTA format
blastdbcmd -db refseq_rna -entry nm_000249 -out test_query.fa

# blastn example
blastn -query test_query.fa -db refseq_rna -task blastn -dust no -outfmt 7 -num_alignments 2 -
num_descriptions 2

# blastp example
blastp -query input.txt -db nr  -task blastp  -num_descriptions 10 -num_alignments 10 -out protein.txt

## AUGUSTUS installation ##
# Append to the path so that Augustus programs will run from any directory
PATH=$PATH:$HOME/Gene_Prediction_Programs/Augustus/augustus.2.5.5/bin
export PATH
echo $PATH

# Alternatively can add the following two lines to .bash_profile
PATH=$PATH:$HOME/Gene_Prediction_Programs/Augustus/augustus.2.5.5/bin
export PATH

# Add the following line to .bashrc in home directory
export AUGUSTUS_CONFIG_PATH=$HOME/Gene_Prediction_Programs/augustus.2.5.5/config

## Example of how to run Augustus
augustus --species=fly --predictionStart=7000001 --predictionEnd=7500000 chr2R.fa > sg.abinitio.gff
```

```
## Information on the GFF format obtained from:
http://www.sanger.ac.uk/resources/software/gff/

## Download the program getAnnoFasta.pl
lwp-download http://augustus.gobics.de/binaries/scripts/getAnnoFasta.pl getAnnoFasta.pl

# Extract the Protein sequences from the gff file
perl getAnnoFasta.pl  mytest.abinitio.gff

## How to train Augustus can be found at:
http://molecularevolution.org/molevolfiles/exercises/augustus/training.html

#setup at folder for the model parameters in /home/Augustus/augustus.2.5.5/config/species
perl new_species.pl --species=toxoplasma_250

# Train the model
etraining --species=toxoplasma_250 aug_train_250.gb

# Make the Augustus predictions
augustus --species=toxoplasma_250 /home/ME49_Chromosomes/TGME49_chrVIIb.txt >
TGME49_chrVIIb_250.gff

augustus --species=human /home/ME49_Chromosomes/TGME49_chrVIIb.txt > aug_human.gff
augustus --species=toxoplasma /home/ME49_Chromosomes/TGME49_chrVIIb.txt > aug_default.gff

## SNAP installation ##

# Download snap
lwp-download http://homepage.mac.com/iankorf/snap-2010-07-28.tar.gz

# Decompress tar.gz file
gunzip snap-2010-07-28.tar.gz
tar -xvf nap-2010-07-28.tar

# Add to the file .bashrc in home directory
export ZOE=$HOME/Gene_Prediction_Programs/SNAP/snap

# Add path to the file .bash_profile
:$HOME/Gene_Prediction_Programs/SNAP/snap

# SNAP options
SNAP - Semi-HMM-based Nucleic Acid Parser (version 2006-07-28)

usage: snap [options] <HMM file> <FASTA file> [options]
options:
  -help          report useful information
  -lcmask        treat lowercase as N
  -plus          predict on plus strand only
  -minus         predict on minus strand only
  -gff           output annotation as GFF
  -ace           output annotation as ACED
  -quiet         do not send progress to STDERR
  -aa <file>     create FASTA file of proteins
  -tx <file>     create FASTA file of transcripts
  -xdef <file>   external definitions
  -name <string>  name for the gene [default snap]

# Example input
./snap HMM/worm DNA/worm.dna.gz
```

```
./snap A.thaliana.hmm thale.dna

# ***** Create SNAP training set *****

# create a .zff file from the exons.txt file
perl create_zff.pl

# Rename the exons.zff file to exons.ann
# Rename DNA sequence file (e.g.TGME49_chrVIIb.txt) with extention .dna
(e.g.TGME49_chrVIIb.dna)

# Run a program called fathom
fathom At.ann At.dna -gene-stats
fathom exons.ann seq.dna -gene-stats
fathom exons.ann seq.dna -validate
fathom exons.ann seq.dna -categorize 1000

# There will be up to 1000 bp on either side of the genes. The files are:

   alt.ann, alt.dna (genes with alternative splicing)
   err.ann, err.dna (genes that have errors)
   olp.ann, olp.dna (genes that overlap other genes)
   wrn.ann, wrn.dna (genes with warnings)
   uni.ann, uni.dna (single gene per sequence)

# Convert the uni genes to plus stranded with the command:
fathom uni.ann uni.dna -export 1000 -plus

# The above generates 4 new files:
   export.aa   proteins corresponding to each gene
   export.ann  gene structure on the plus strand
   export.dna  DNA of the plus strand
   export.tx   transcripts for each gene

#Create a new directory for the parameter estimations
   mkdir params
   cd params
   forge ../export.ann ../export.dna
   cd ..

# build an HMM
perl hmm-assembler.pl  split_seq.dna params > toxo.hmm

# Example of how to run SNAP with training set
snap toxo.hmm TGME49_chrVIIb.txt -aa TGME49_chrVIIb.aa -tx TGME49_chrVIIb.tx -gff >
TGME49_chrVIIb.gff

## GeneMark installation ##

# Downloaded from http://exon.gatech.edu/license_download.cgi

# WebPage on http://exon.gatech.edu/

# Decompress tar.gz file
gunzip gm_es_bp_linux64_v2.3d.tar.gz
tar -xvf gm_es_bp_linux64_v2.3d.tar

# Rename gm_es_bp_linux64_v2.3d gmes

# Extract the key file (licence file)
```

```
tar -xvf gm_key_64.tar

# Move the file gm_key to home directory and rename to .gm_key

# re-format input file into a FASTA format
perl re_format_fasta.pl --out fasta_chrVIIb.txt --trace trace.txt  TGME49_chrVIIb.txt

# To run GeneMark-ES
perl /home/sgoodswe/Gene_Prediction_Programs/GeneMark/gmes/gm_es.pl fasta_chrVIIb.txt --BP
OFF

#To run GenMark_HMM

#GenMark.hmm options
GeneMark.hmm eukaryotic, version bp 3.9d April 16, 2009

Usage: gmhmme3 [options] <sequence file>

  required parameters:
   -m <model file>

  optional parameters:
   -o <output file>
   -p write protein translation
   -b <output file> output statistics of predicted introns
   -d <file name> provide input for GeneMark.hmm plus
   -s <string> sequence tag in GFF output format
   -f <format> output prediction in [lst|gff3|gtf] format; default [lst]


#Example of how to run GeneMark.HMM
$HOME/Gene_Prediction_Programs/GeneMark/genemark_hmm/gmhmme3 fasta_chrVIIb.txt -m
$HOME/Gene_Prediction_Programs/GeneMark/genemark_hmm/t_gondii_05.hmm3.mod -p

## GlimmerHMM

# Download Glimmer
lwp-download ftp://ftp.cbcb.umd.edu/pub/software/glimmerhmm/GlimmerHMM-3.0.1.tar.gz

# Decompress tar.gz file
gunzip GlimmerHMM-3.0.1.tar.gz
tar -xvf GlimmerHMM-3.0.1.tar

#add to .bash_profile
/home/sgoodswe/Gene_Prediction_Programs/Glimmer/bin

# To train GlimmerHMM, first run make in the train directory.
# Read readme.train from GlimmerHMM/train. After creating training directory you can use it at the -#
d option with GlimmerHMM.

# trainGlimmerHMM <mfasta_file> <exon_file> [optional_parameters]

perl $HOME/Gene_Prediction_Programs/Glimmer/train/trainGlimmerHMM seq_genes_chr_VIIb.txt
glim_exons.txt

#Glimmer options

USAGE:  glimmerhmm_linux <genome1-file> <training-dir-for-genome1> [options]
Options:
-p file_name     If protein domain searches are available, read them from file file_name
```

-d dir_name Training directory is specified by dir_name (introduced for compatibility with earlier versions)
-o file_name Print output in file_name; if n>1 for top best predictions, output is in file_name.1, file_name.2, file_name.n f-n n Print top n best predictions
-g Print output in gff format
-v Don't use svm splice site predictions
-f Don't make partial gene predictions
-h Display the options of the program

#how to run Glimmer
glimmerhmm_linux $HOME/toxo_predictions/ME49_Chromosomes/TGME49_chrVIIb.txt -d $HOME/Gene_Prediction_Programs/Glimmer/trained_dir/toxoplasma -o glimmer_results.txt

glimmerhmm_linux $HOME/toxo_predictions/ME49_Chromosomes/TGME49_chrVIIb.txt -d $HOME/Gene_Prediction_Programs/Glimmer/trained_dir/toxoplasma -o glimmer_results.gff -g

Examples of gene finder execution

Augustus

augustus --species=toxoplasma TGME49_chrVIIb.txt > aug_TGME49_chrVIIb.gff

perl getAnnoFasta.pl aug_TGME49_chrVIIb.gff

blastp -query aug_TGME49_chrVIIb.aa -db nr -task blastp -outfmt "10 qseqid qgi qacc qaccver qlen sseqid sallseqid sgi sallgi sacc saccver sallacc slen evalue bitscore score pident nident mismatches positive " -num_descriptions 10 -out blast_aug_ME49_chrVIIb.txt

SNAP

snap toxo.hmm TGME49_chrVIIb.txt -aa snap_TGME49_chrVIIb.aa -tx snap_TGME49_chrVIIb.tx -gff > snap_TGME49_chrVIIb.gff

blastp -query snap_TGME49_chrVIIb.aa -db nr -task blastp -outfmt "10 qseqid qgi qacc qaccver qlen sseqid sallseqid sgi sallgi sacc saccver sallacc slen evalue bitscore score pident nident mismatches positive " -num_descriptions 10 -out blast_snap_ME49_chrVIIb.txt

GenMark.HMM

$HOME/Gene_Prediction_Programs/GeneMark/genemark_hmm/gmhmme3 TGME49_chrVIIb.txt -m $HOME/Gene_Prediction_Programs/GeneMark/genemark_hmm/t_gondii_05.hmm3.mod -p

perl get_prot_seq.pl [This script needed to be written to get sequences from bottom of file TGME49_chrVIIb.txt.lst

blastp -query gm_TGME49_chrVIIb.aa -db nr -task blastp -outfmt "10 qseqid qgi qacc qaccver qlen sseqid sallseqid sgi sallgi sacc saccver sallacc slen evalue bitscore score pident nident mismatches positive " -num_descriptions 10 -out blast_gm_ME49_chrVIIb.txt

Glimmer.HMM

glimmerhmm_linux $HOME/toxo_predictions/ME49_Chromosomes/TGME49_chrVIIb.txt -d $HOME/Gene_Prediction_Programs/Glimmer/trained_dir/toxoplasma -o gl_TGME49_chrVIIb.gff -g
